# Joint assessment of white blood cell-to-HDL cholesterol ratio and waist-to-height-hemoglobin A1c for cardiometabolic multimorbidity risk: a prospective cohort study and cross-sectional study

**DOI:** 10.3389/fnut.2026.1855163

**Published:** 2026-06-30

**Authors:** Xixi Han, Shuai Hou, Jingwen Kong, Baike Liu, Bingbing Li, Chao Wei

**Affiliations:** 1 Integrated Traditional Chinese and Western Medicine Clinical Laboratory, College of Integrated Traditional Chinese and Western Medicine, Jining Medical University, Jining, China; 2 Dongzhimen Hospital, Beijing University of Chinese Medicine, Beijing, China; 3 Affiliated Hospital of Jining Medical University, Jining, China

**Keywords:** cardiometabolic multimorbidity, CHARLS, combined biomarker, waist-to-height-hemoglobin A1c, white blood cell-to-HDL cholesterol ratio

## Abstract

**Background:**

The pathogenesis of cardiometabolic multimorbidity (CMM) is complex and involves multiple interconnected pathological processes. Biomarkers that comprehensively reflect these mechanisms, particularly when used in combination, are critical for early identification of individuals at risk of CMM. Therefore, this study aimed to investigate the association of the white blood cell-to-HDL cholesterol ratio (WHR) and waist-to-height-hemoglobin A1c (WHH) with CMM risk, and to evaluate their combined predictive value.

**Methods:**

Two cohorts were established using data from the China Health and Retirement Longitudinal Study (CHARLS). Participants were categorized into four groups based on median cutoffs of WHH and WHR. Kaplan–Meier curves, Cox regression, restricted cubic spline (RCS) analysis, mediation analysis, receiver operating characteristic (ROC) curves, net reclassification improvement (NRI), integrated discrimination improvement (IDI), interaction analysis, and weighted quantile sum (WQS) regression were applied to assess the association, predictive performance, and underlying mechanisms linking WHH and WHR to CMM risk. Additionally, a cross-sectional dataset from the U. S. National Health and Nutrition Examination Survey (NHANES) was included for external validation, to confirm the stability and reliability of the core findings.

**Results:**

WHH and WHR were significantly and independently associated with an increased risk of CMM. The hazard ratio (HR) for CMM in the high WHH + high WHR group was 3.28 (95% CI: 2.28–4.73; *p* < 0.001). RCS analysis revealed a nonlinear association between WHR and CMM risk (*p* for nonlinearity = 0.043). The combined WHH–WHR model showed superior predictive performance, as reflected by higher NRI and IDI values. Bidirectional mediating effects were observed between WHH and WHR. WQS regression identified waist circumference, glycated hemoglobin (HbA1c), and white blood cell (WBC) count as the major contributing components. Sensitivity analyses confirmed the robustness of the results. External validation using the NHANES cros-sectional data yielded findings consistent with those from the CHARLS cohort, demonstrating satisfactory predictive performance.

**Conclusion:**

The combination of WHH and WHR is significantly associated with elevated CMM risk and may serve as an economical and convenient biomarker.

## Graphical abstract

**Figure:**
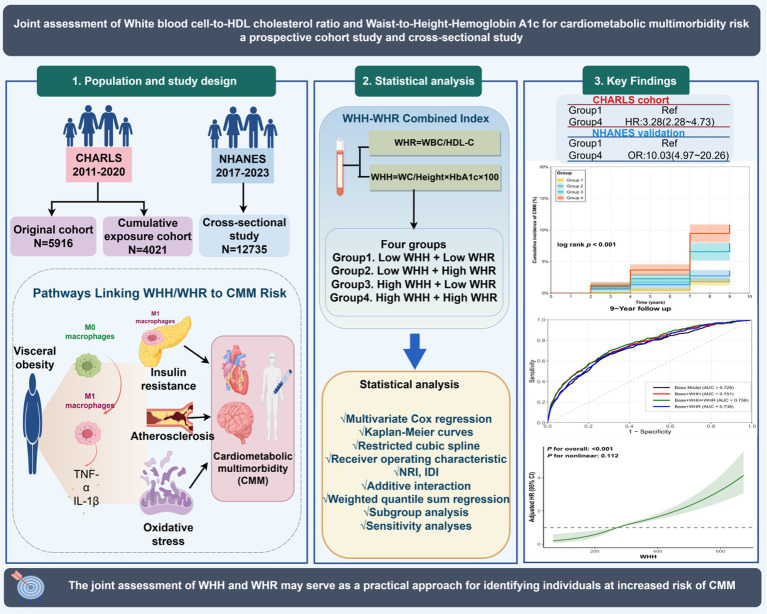

## Introduction

Cardiometabolic multimorbidity (CMM) is defined as the simultaneous occurrence of at least two cardiovascular and metabolic diseases, including diabetes, heart disease, and stroke, representing one of the most severe forms of chronic disease comorbidity (1). With rapid population aging, the prevalence of CMM continues to rise, closely linked to increased healthcare costs, reduced quality of life, shortened life expectancy, and doubled mortality risk (2–4). Therefore, early identification and intervention for individuals at high risk of CMM carry important clinical and public health implications.

The pathogenesis of CMM involves multiple interconnected pathophysiological mechanisms. Adipose tissue, skeletal muscle, pancreas, and liver form complex metabolic networks. Pathological processes including central obesity, insulin resistance (IR), lipid dysregulation, chronic low-grade inflammation, and endothelial dysfunction interact synergistically, accelerating metabolic derangements and contributing to CMM onset (4–7). Accordingly, several biomarkers, such as the C-reactive protein-triglyceride-glucose index (CTI), triglyceride-glucose (TyG)-related indices, estimated glucose disposal rate (eGDR), and atherogenic index of plasma (AIP), have been developed to assess cardiometabolic risk (8–10). However, these indices fail to comprehensively integrate multi-dimensional information on obesity, inflammation, and glucolipid metabolism, and their efficacy in predicting CMM risk remains insufficiently validated.

As an integrated inflammatory–lipid biomarker, the white blood cell-to-high-density lipoprotein cholesterol ratio (WHR) comprehensively reflects systemic inflammatory burden, lipid dysregulation, and endothelial dysfunction, with validated associations with ischemic heart disease and cardiovascular disease risk (11, 12). Waist-to-height-hemoglobin A1c (WHH) is calculated as the product of the waist-to-height ratio (WHtR) and glycated hemoglobin (HbA1c), integrating two key risk factors: central obesity and long-term glycemic exposure. WHH has been shown to predict diabetes risk more effectively than TyG and METS-IR (13). Notably, despite their distinct focuses, WHR and WHH share fundamental pathophysiological links. On one hand, obesity and hyperglycemia induce pro-inflammatory M1 polarization and impaired lipid metabolism in adipose tissue macrophages (ATMs), altering WHR levels (14, 15). On the other hand, chronic inflammation reduces insulin sensitivity, exacerbates IR, elevates HbA1c, and promotes abdominal obesity, ultimately influencing WHH (16, 17). Existing evidence indicates that macrophage counts in obese individuals predict HbA1c concentrations (18). Thus, combined use of WHR and WHH may provide more comprehensive information for CMM risk prediction. However, the predictive value of their combined application and the impact of two-time-point cumulative exposure on CMM risk remain unclear.

Accordingly, using nationally representative data from the China Health and Retirement Longitudinal Study (CHARLS), this study constructed baseline and cumulative exposure cohorts to systematically evaluate the association of WHR and WHH, as well as their combined model, with CMM risk. We further explored their combined predictive value, mediating effects, and interactions, aiming to identify novel biomarkers for early risk stratification of CMM. Additionally, cross-sectional data from the U. S. National Health and Nutrition Examination Survey (NHANES) were used for external validation to confirm the robustness of the core findings.

## Methods

### Study design and population

The cohort was derived from CHARLS, a nationwide, population-based prospective study designed to collect social, economic, and health-related information among Chinese adults aged 45 years and older (19). All participants provided written informed consent, and the study protocol was approved by the Biomedical Ethics Committee of Peking University (IRB00001052-11015).

**Figure 1:**
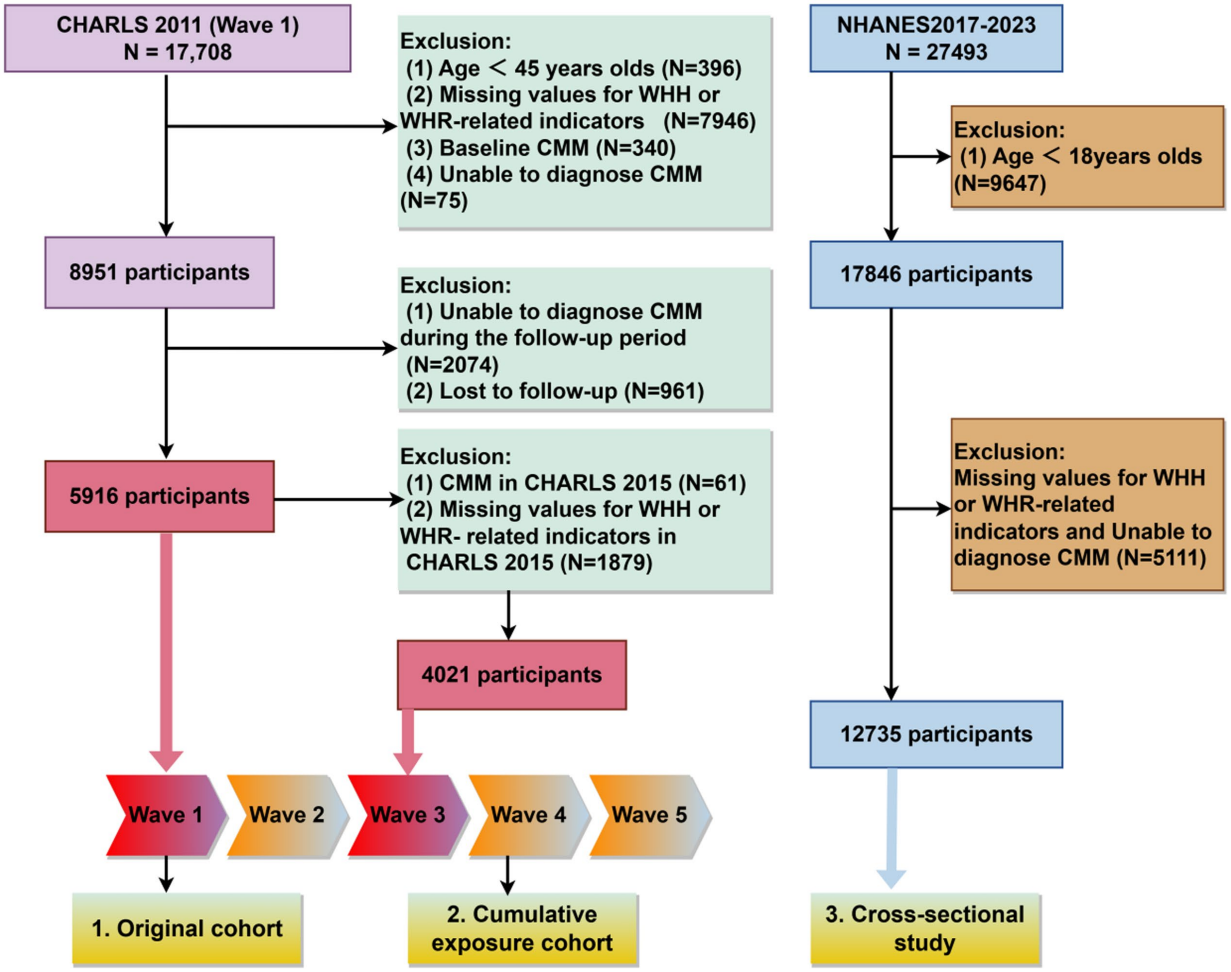
Flowchart of participants included in this study.

The CHARLS baseline survey was conducted in 2011–2012, with follow-up assessments in 2013, 2015, 2018, and 2020. Participants were excluded if they were aged ≤45 years, had missing data for either WHH or WHR, had prevalent CMM at baseline, or lacked complete information for CMM diagnosis at baseline or during follow-up. A total of 5,916 participants were included in the primary analysis. To evaluate cumulative exposure, a secondary cohort was further constructed by additionally excluding participants with missing WHH/WHR data in 2015 or those who developed CMM by 2015, yielding a final sample of 4,021 participants. Cross-sectional data were obtained from the 2017–2023 NHANES,^1^ including 12,735 adults aged 20–80 years with complete demographic, examination, and laboratory data (Figure 1).

### Calculation of exposure variables

WHH was calculated as (13):

WHH=(waist circumference[cm]/height[cm])×HbA1c×100

WHR was calculated as (11):

$\begin{array}{l} \mathrm{WHR}=\text{white blood cell count}\;(10^{9}/L)/\mathrm{HDL}‐\text{cholesterol}\;\\ \left(\text{mmol}/L\right) \end{array}$

Cumulative WHH (cuWHH) and cumulative WHR (cuWHR) were computed to reflect two-time-point cumulative exposure using established formulas (20, 21).

$\text{cuWHH}=[({\mathrm{WHH}}_{2012}+{\mathrm{WHH}}_{2015})/2]\times (2015–2012)$

$\text{cuWHR}=[({\mathrm{WHR}}_{2012}+{\mathrm{WHR}}_{2015})/2]\times (2015–2012)$

### Definition and evaluation of outcomes

The primary outcome was incident CMM, defined as the simultaneous occurrence of at least two of the following: diabetes, stroke, and heart disease (22–24). These conditions share common pathological mechanisms, including obesity, inflammation, and insulin resistance, and their co-occurrence is associated with a substantially higher mortality risk than individual diseases, imposing a heavy disease burden on middle-aged and older populations. The diagnostic criteria for diabetes include at least one of the following: (1) a physician-reported diagnosis of diabetes or hyperglycemia, (2) current treatment with western medicine, traditional Chinese medicine, or insulin for glycemic control, (3) HbA1c ≥ 6.5%, or (4) fasting plasma glucose (FPG) > 125 mg/dL (25). The diagnostic criterion for stroke is a physician-reported diagnosis or a history of stroke treatment (26). Heart disease is defined as a physician-diagnosed heart attack, coronary heart disease, angina, congestive heart failure, or other cardiac conditions, or current use of cardiovascular medications (27). CMM was defined as the concurrent presence of ≥2 conditions in any follow-up wave. Follow-up duration was calculated from the 2011 baseline to the year of CMM onset (for cases) or the last follow-up (for non-cases) (21, 28).

### Covariates

Covariates included age, sex, marital status, education, smoking, alcohol consumption, height, weight, waist circumference, body mass index, systolic blood pressure, diastolic blood pressure, chronic disease status, medication use, and laboratory parameters. Hypertension is defined as meeting any of the following criteria: a physician-diagnosed history, current antihypertensive treatment, systolic blood pressure (SBP) ≥ 140 mmHg, or diastolic blood pressure (DBP) ≥ 90 mmHg (29, 30). Dyslipidemia is defined as a physician-reported diagnosis, ongoing lipid-lowering treatment, or abnormal lipid parameters (TC ≥ 240 mg/dL, TG ≥ 150 mg/dL, LDL-C ≥ 160 mg/dL, HDL-C < 40 mg/dL) (28).

### Handling of missing data

Missing data (most frequent for cystatin C and alcohol consumption) were imputed using multiple imputation by chained equations (MICE) with five imputations. Pooled estimates were obtained using Rubin’s rules (Supplementary Tables S1, S2).

### Statistical analysis

Since no established clinical cutoffs exist for WHH and WHR, participants were stratified into high and low groups based on median values, yielding four categories: low WHH + low WHR, low WHH + high WHR, high WHH + low WHR, and high WHH + high WHR. The cumulative exposure cohort was stratified identically. Continuous variables are presented as mean ± standard deviation (normal distribution) or median (interquartile range) (non-normal distribution). Categorical variables are reported as frequencies (%). Group differences were assessed using Student’s t-test, Mann–Whitney U test, or chi-square (*χ*^2^) test.

Kaplan–Meier curves and log-rank tests were used to compare cumulative CMM incidence across groups. Multivariate Cox proportional hazards models were applied to evaluate the association between the combined WHH–WHR model and CMM risk, with the low WHH + low WHR group as the reference. Three sequential models were constructed. Model I did not adjust for variables. Model II adjusted for age, gender, marital status, education. Model III adjusted for age, gender, marital status, education, smoking, drinking, SBP, DBP, UA, Scr, BUN, CRP, Cystatin C, Hb, hypertension, chronic lung diseases, liver disease, kidney disease, depression, use of antihypertensive drugs and use of hypoglycemic agents. Multicollinearity diagnostics and Schoenfeld residual tests were performed (Supplementary Tables S3–S6). Restricted cubic spline (RCS) regression with four knots was used to explore nonlinear relationships between WHH/WHR and CMM risk. Piecewise Cox regression was applied for significant nonlinear associations, with optimal cutoffs determined via likelihood ratio tests.

Receiver operating characteristic (ROC) curves were used to assess predictive performance at 4, 7, and 9 year follow-up. Area under the curve (AUC) differences were evaluated using the DeLong test. Net reclassification improvement (NRI) and integrated discrimination improvement (IDI) were calculated to quantify incremental predictive value. Bidirectional mediation analysis was performed to examine mediating effects on the association between WHH, WHR, and CMM risk, with results reported as the proportion of the indirect effect relative to the total effect. Additive interactions were assessed using the synergy index (SI), attributable proportion due to interaction (AP), and relative excess risk due to interaction (RERI), with 95% CIs calculated via the Mover method. Weighted quantile sum (WQS) regression was used to estimate the contribution weights of WHH and WHR components to CMM risk.

Subgroup analyses were stratified by key variables, and heterogeneity was tested via interaction terms. Sensitivity analyses were conducted to verify result robustness. Cross-sectional analyses employed weighted multivariable logistic regression and ROC curves to account for sampling bias. All analyses were performed using R software (v4.5.1), with *p* < 0.05 considered statistically significant. Key packages included survival, rms, riskRegression, nricens, survIDINRI, CMAverse, interactionR, and gWQS.

## Results

### Baseline characteristics

The baseline characteristics stratified by WHH–WHR categories are presented in Table 1. A total of 5,916 participants were included, consisting of 3,233 females and 2,683 males, with a mean age of 57.8 ± 8.6 years. During the follow-up period from 2011 to 2020, 355 participants (5.96%) developed CMM. The incidence rates increased sequentially across the four groups, at 2.2, 3.5, 7.9, and 10.8%, respectively. Compared with the low WHH + low WHR group, the other three groups had significantly higher levels of BMI, SBP, DBP, WC, weight, PLT, WBC, TG, TC, LDL-C, HbA1c, FBG, UA, and CRP. The prevalence of chronic diseases (hypertension, diabetes, dyslipidemia) and the use of lipid-lowering, antihypertensive, and hypoglycemic medications showed a significant upward trend, while HDL-C levels decreased significantly (all *p* < 0.001). As shown in Supplementary Table S7, the cumulative exposure cohort included 4,021 participants, with 181 individuals (4.5%) developing CMM during the 5-year follow-up; the highest incidence rate (8.1%) was observed in the high cuWHH + high cuWHR group. Similar findings were observed in the cross-sectional study (Table 2).

**Table 1:** Baseline characteristics grouped according to the WHH-WHR combination (CHARLS cohort).

Variables	Total	Group 1	Group 2	Group 3	Group 4	*p*
n	5,916	1851	1,212	1,235	1,618	
CMM	355 (6.0)	40 (2.2)	43 (3.5)	97 (7.9)	175 (10.8)	< 0.001
Age, years	57.8 ± 8.6	57.8 ± 8.6	56.5 ± 8.3	58.9 ± 8.5	58.0 ± 8.6	< 0.001
Gender						< 0.001
Female	3,233 (54.6)	883 (47.7)	466 (38.4)	887 (71.8)	997 (61.6)	
Male	2,683 (45.4)	968 (52.3)	746 (61.6)	348 (28.2)	621 (38.4)	
Marital status						0.031
Married	5,354 (90.5)	1,666 (90)	1,110 (91.6)	1,096 (88.7)	1,482 (91.6)	
Unmarried	562 (9.5)	185 (10)	102 (8.4)	139 (11.3)	136 (8.4)	
Education						< 0.001
Primary education	4,130 (69.8)	1,300 (70.2)	787 (64.9)	927 (75.1)	1,116 (69)	
Secondary education	1,641 (27.7)	509 (27.5)	395 (32.6)	282 (22.8)	455 (28.1)	
Higher education	145 (2.5)	42 (2.3)	30 (2.5)	26 (2.1)	47 (2.9)	
Smoking	2,202 (37.2)	774 (41.8)	628 (51.8)	287 (23.2)	513 (31.7)	< 0.001
Drinking	1,319 (22.3)	462 (25)	284 (23.4)	224 (18.1)	349 (21.6)	< 0.001
BMI, kg/m^2^	23.8 ± 12.8	21.3 ± 2.7	22.1 ± 3.0	25.0 ± 13.7	26.8 ± 20.6	< 0.001
SBP, mmHg	128.4 ± 20.4	124.5 ± 19.4	125.6 ± 19.0	129.7 ± 20.5	134.0 ± 21.1	< 0.001
DBP, mmHg	75.1 ± 11.8	73.0 ± 11.7	73.9 ± 11.4	75.4 ± 11.5	78.2 ± 11.7	< 0.001
Waist circumference, cm	83.7 ± 12.4	76.3 ± 11.2	78.0 ± 12.4	88.8 ± 7.9	92.6 ± 8.2	< 0.001
Height, cm	157.8 ± 9.1	158.9 ± 8.2	160.4 ± 8.3	154.7 ± 8.7	156.8 ± 10.1	< 0.001
Weight, kg	58.5 ± 11.0	54.1 ± 9.3	57.0 ± 9.9	59.2 ± 10.2	64.3 ± 11.6	< 0.001
PLT, 10^9^/L	211.8 ± 71.4	199.3 ± 65.4	220.6 ± 77.0	203.4 ± 66.4	225.9 ± 73.7	< 0.001
WBC, 10^9^/L	6.2 ± 1.8	5.2 ± 1.2	7.4 ± 1.9	5.3 ± 1.1	7.2 ± 1.8	< 0.001
TG, mmol/L	1.2 (0.85, 1.77)	0.91 (0.71, 1.24)	1.1 (0.81, 1.48)	1.31 (0.92, 1.89)	1.64 (1.17, 2.46)	< 0.001
HDL-C, mmol/L	1.32 ± 0.4	1.58 ± 0.37	1.5 ± 0.36	1.13 ± 0.28	1.06 ± 0.27	< 0.001
TC, mmol/L	5 ± 1.01	4.88 ± 0.92	5.23 ± 0.96	4.77 ± 0.98	5.15 ± 1.08	< 0.001
LDL-C, mmol/L	3 ± 0.9	2.89 ± 0.84	3.23 ± 0.88	2.88 ± 0.88	3.08 ± 0.98	< 0.001
HbA1c, %	5.3 ± 0.8	5.0 ± 0.3	4.9 ± 0.4	5.5 ± 0.8	5.7 ± 1.0	< 0.001
FBG, mg/dL	109.6 ± 35.2	100.7 ± 16.8	103.8 ± 21.8	111.2 ± 36.5	122.9 ± 50.3	< 0.001
UA, mg/dL	4.4 ± 1.2	4.2 ± 1.2	4.5 ± 1.3	4.2 ± 1.1	4.6 ± 1.3	< 0.001
Scr, mg/dL	0.8 ± 0.2	0.8 ± 0.2	0.8 ± 0.2	0.7 ± 0.2	0.8 ± 0.2	< 0.001
BUN, mg/dL	15.7 ± 4.3	15.9 ± 4.3	15.5 ± 4.3	15.7 ± 4.3	15.5 ± 4.4	0.038
CRP, mg/dL	0.9 (0.5, 2.0)	0.6 (0.4, 1.2)	1.0 (0.5, 2.3)	0.9 (0.5, 1.7)	1.4 (0.8, 2.9)	< 0.001
Cystatinc, mg/dL	1.0 ± 0.2	1.0 ± 0.2	1.0 ± 0.2	1.0 ± 0.2	1.0 ± 0.3	< 0.001
Hb, g/dL	14.4 ± 2.2	14.1 ± 2.3	14.5 ± 2.1	14.2 ± 2.3	14.6 ± 2.2	< 0.001
Hypertension	2099 (35.5)	485 (26.2)	331 (27.3)	485 (39.3)	798 (49.3)	< 0.001
Diabetes	923 (15.6)	137 (7.4)	119 (9.8)	209 (16.9)	458 (28.3)	< 0.001
Heart disease	156 (2.6)	53 (2.9)	33 (2.7)	34 (2.8)	36 (2.2)	0.673
Dyslipidemia	1,143 (19.3)	219 (11.8)	156 (12.9)	259 (21)	509 (31.5)	< 0.001
Stroke	34 (0.6)	10 (0.5)	3 (0.2)	5 (0.4)	16 (1)	0.051
Chronic lung diseases	495 (8.4)	156 (8.4)	102 (8.4)	111 (9)	126 (7.8)	0.719
Liver disease	203 (3.4)	69 (3.7)	35 (2.9)	51 (4.1)	48 (3)	0.217
Cancer	56 (0.9)	22 (1.2)	9 (0.7)	7 (0.6)	18 (1.1)	0.254
Kidney disease	352 (5.9)	132 (7.1)	68 (5.6)	71 (5.7)	81 (5)	0.057
Depression	63 (1.1)	17 (0.9)	17 (1.4)	17 (1.4)	12 (0.7)	0.218
Use of lipid-lowering drugs	1,169 (19.8)	230 (12.4)	159 (13.1)	270 (21.9)	510 (31.5)	< 0.001
Use of antihypertensive drugs	851 (14.4)	142 (7.7)	114 (9.4)	185 (15)	410 (25.3)	< 0.001
Use of hypoglycemic agents	170 (2.9)	9 (0.5)	5 (0.4)	40 (3.2)	116 (7.2)	< 0.001

Normally distributed data are presented as mean ± SD, non-normally distributed data are presented as medians (interquartile range). Categorical variables are presented as frequency (%). CMM, Cardiometabolic multimorbidity; BMI, Body mass index; SBP, Systolic blood pressure; DBP, Diastolic blood pressure; PLT, Platelets; WBC, White blood cell; TG, Triglycerides; HDL-C, High Density Lipoprotein-Cholesterol; TC, Total cholesterol; LDL-C, Low-density lipoprotein cholesterol; HbA1c, Glycated hemoglobin; FPG, Fasting plasma glucose; UA, Serum uric acid; Scr, Serum Creatinine; BUN, Blood Urea Nitrogen; CRP, c-reactive protein; Hb, Hemoglobin.

**Table 2:** Baseline characteristics grouped according to the WHH-WHR combination (NHANES).

Variables	Total	Group 1	Group 2	Group 3	Group 4	*p*
*n*	12,735	3,994	2,374	2,376	3,991	
CMM	435 (3.4%)	20 (0.5%)	13 (0.5%)	127 (5.3%)	275 (6.9%)	< 0.001
Age, years	54 (37, 66)	48 (33, 63)	41 (30, 57)	63 (53, 71)	57 (42, 68)	< 0.001
Gender, *n* (%)						< 0.001
Male	6,030 (47.3%)	1738 (43.5%)	1,476 (62.2%)	801 (33.7%)	2015 (50.5%)	
Female	6,705 (52.7%)	2,256 (56.5%)	898 (37.8%)	1,575 (66.3%)	1976 (49.5%)	
Education, *n* (%)						< 0.001
Less than 9th grade	790 (6.2%)	117 (2.9%)	133 (5.6%)	204 (8.6%)	336 (8.4%)	
9-11th grade	1,203 (9.4%)	262 (6.6%)	253 (10.7%)	230 (9.7%)	458 (11.5%)	
High school graduate	2,883 (22.6%)	748 (18.7%)	570 (24%)	570 (24%)	995 (24.9%)	
Some college or AA degree	4,053 (31.8%)	1,214 (30.4%)	719 (30.3%)	747 (31.4%)	1,373 (34.4%)	
College graduate or above	3,795 (29.8%)	1,649 (41.3%)	698 (29.4%)	623 (26.2%)	825 (20.7%)	
Marital status, *n* (%)						< 0.001
Married	7,324 (57.5%)	2,281 (57.1%)	1,387 (58.4%)	1,296 (54.5%)	2,360 (59.1%)	
Divorced	2,935 (23%)	784 (19.6%)	381 (16%)	773 (32.5%)	997 (25%)	
Widowed	2,464 (19.3%)	924 (23.1%)	605 (25.5%)	304 (12.8%)	631 (15.8%)	
Race, *n* (%)						< 0.001
Non-Hispanic White	1,257 (9.9%)	273 (6.8%)	250 (10.5%)	213 (9%)	521 (13.1%)	
Non-Hispanic Black	1,325 (10.4%)	354 (8.9%)	292 (12.3%)	226 (9.5%)	453 (11.4%)	
Mexican American	5,817 (45.7%)	1886 (47.2%)	1,108 (46.7%)	980 (41.2%)	1843 (46.2%)	
Other Hispanic	2,481 (19.5%)	821 (20.6%)	288 (12.1%)	694 (29.2%)	678 (17%)	
Other Race	1855 (14.6%)	660 (16.5%)	436 (18.4%)	263 (11.1%)	496 (12.4%)	
Current smoker, n (%)	364 (2.9%)	179 (4.5%)	46 (1.9%)	72 (3%)	67 (1.7%)	< 0.001
Regular drinker, n (%)	1,657 (13%)	404 (10.1%)	495 (20.9%)	172 (7.2%)	586 (14.7%)	< 0.001
Albumin, g/dL	4.1 (3.9, 4.3)	4.2 (4, 4.4)	4.2 (4, 4.4)	4 (3.8, 4.2)	4 (3.8, 4.2)	< 0.001
ALT, IU/L	19 (16, 24)	20 (17, 24)	19 (16, 24)	20 (16, 24)	19 (16, 24)	< 0.001
AST, IU/L	76 (63, 93)	69 (57, 84)	76 (63, 91)	80 (66, 98)	83 (69, 100)	< 0.001
BUN, mmol/L	14 (11, 17)	14 (11, 17)	13 (11, 17)	15 (12, 19)	14 (12, 18)	< 0.001
Scr, umol/L	0.8 (0.7, 1)	0.8 (0.7, 1)	0.9 (0.7, 1)	0.8 (0.7, 1)	0.8 (0.7, 1)	< 0.001
SUA, mg/dL	5.2 (4.3, 6.2)	4.7 (3.9, 5.7)	5.2 (4.4, 6.2)	5.2 (4.3, 6.2)	5.6 (4.7, 6.7)	< 0.001
hs-CRP, mg/L	1.9 (0.8, 4.3)	0.9 (0.5, 2)	1.6 (0.8, 3.4)	2.4 (1.1, 4.7)	3.6 (1.8, 7)	< 0.001

CMM, Cardiometabolic multimorbidity; ALT, Alanine Aminotransferase; AST, Aspartate Aminotransferase; BUN, Blood Urea Nitrogen; Scr, Serum Creatinine; SUA, Serum uric acid; hs-CRP, High-sensitivity C-reactive protein.

### Association between WHH, WHR and their combined models with CMM

Cox proportional hazards models were applied to examine the association of baseline and cumulative WHH and WHR, as well as their combined groups, with CMM risk (Table 3). After adjustment for Model III, both WHH and WHR were significantly and positively associated with elevated CMM risk, with consistent findings observed for cumulative exposure analyses (all *p* < 0.001). Combined group analysis revealed a clear dose–response relationship for CMM risk across categories (*p* for trend < 0.001). After full covariate adjustment, compared with the low WHH + low WHR group (Group 1), the high WHH + high WHR group (Group 4) exhibited a 3.28-fold higher risk of CMM (HR = 3.28, 95% CI: 2.28–4.73, *p* < 0.001). Similarly, the high cuWHH + high cuWHR group had a 3.37-fold higher risk than the low cuWHH + low cuWHR group (HR = 3.37, 95% CI: 2.01–5.65, *p* < 0.001). Separate Cox regression analyses using quartiles of baseline WHH, WHR, cuWHH, and cuWHR also demonstrated significant positive associations with CMM risk and a graded increasing trend (*p* for trend < 0.001; Supplementary Table S8). Consistently, cross-sectional analysis indicated that, in the fully adjusted model, Group 4 was associated with a 10.03-fold higher CMM risk than Group 1 (OR = 10.03, 95% CI: 4.97–20.26, *P* = < 0.001; Table 4).

**Table 3:** Association of WHH, WHR, and their combined groups with CMM in the CHARLS cohorts.

Characteristic	N (Incidence rate)	Model 1		Model 2		Model 3	
		HR (95% CI)	*p* value	HR (95% CI)	*p* value	HR (95% CI)	*p* value
WHH		1.0044 (1.0039–1.0048)	<0.001	1.004 (1.004–1.005)	<0.001	1.0037 (1.003–1.004)	<0.001
WHR	–	1.08 (1.06–1.09)	<0.001	1.09 (1.07–1.11)	<0.001	1.06 (1.04–1.08)	<0.001
WHH-WHR combined groups	–						
Group 1	40 (2.2)	Ref	–	Ref	–	Ref	–
Group 2	43 (3.5)	1.66 (1.08–2.55)	0.022	1.69 (1.1–2.61)	0.017	1.62 (1.05–2.5)	0.031
Group 3	97 (7.9)	3.74 (2.59–5.41)	<0.001	3.21 (2.21–4.67)	<0.001	2.94 (2.01–4.28)	<0.001
Group 4	175 (10.8)	5.25 (3.73–7.41)	<0.001	4.34 (3.06–6.15)	<0.001	3.28 (2.28–4.73)	<0.001
*p* for trend			<0.001		<0.001		<0.001
Cu-WHH		1.0026 (1.0023–1.003)	<0.001	1.0024 (1.002–1.0028)	<0.001	1.002 (1.0015–1.0024)	<0.001
Cu-WHR		1.06 (1.04–1.08)	<0.001	1.07 (1.05–1.09)	<0.001	1.05 (1.03–1.08)	<0.001
Cu-WHH-WHR combined groups							
Group 1	19 (1.6)	Ref	–	Ref	–	Ref	–
Group 2	50 (6)	3.84 (2.26–6.51)	<0.001	3.21 (1.88–5.51)	<0.001	2.82 (1.64–4.85)	<0.001
Group 3	16 (1.9)	1.2 (0.62–2.33)	0.591	1.31 (0.67–2.55)	0.433	1.22 (0.62–2.39)	0.558
Group 4	96 (8.1)	5.23 (3.2–8.56)	<0.001	4.4 (2.67–7.24)	<0.001	3.37 (2.01–5.65)	<0.001
*p* for trend			<0.001		<0.001		<0.001

HR, Hazard ratio; CI, Confidence interval; WHH, Waist-to-Height-Hemoglobin A1c; WHR, White blood cell-to-high-density lipoprotein cholesterol ratio. Group 1: low WHH + low WHR, Group 2: low WHH + high WHR, Group 3: high WHH + low WHR, Group 4: high WHH + high WHR. Model I: unadjusted. Model II: adjusting for age, gender, marital status, education. Model III: adjusting for age, gender, marital status, education, smoking, drinking, SBP, DBP, UA, Scr, BUN, CRP, Cystatinc, Hb, hypertension, chronic lung diseases, liver disease, kidney disease, depression, use of antihypertensive drugs, use of hypoglycemic agents.

**Table 4:** Association of WHH-WHR combined groups with CMM in the NHANES cross-sectional study.

Characteristic	Model 1		Model 2		Model 3	
	OR (95% CI)	*p* value	OR (95% CI)	*p* value	OR (95% CI)	*p* value
WHH-WHR combined groups						
Group1	Ref	–	Ref	–	Ref	–
Group2	1.12 (0.43–2.91)	0.818	1.2 (0.45–3.17)	0.717	1.19 (0.45–3.15)	0.733
Group3	9.79 (4.81–19.93)	<0.001	6.15 (2.98–12.7)	<0.001	5.73 (2.76–11.88)	<0.001
Group4	13.68 (6.96–26.91)	<0.001	10.41 (5.21–20.82)	<0.001	10.03 (4.97–20.26)	<0.001
*p* for trend		<0.001		<0.001		<0.001

OR, Odd ratio; CI, Confidence interval; WHH, Waist-to-Height-Hemoglobin A1c; WHR, White blood cell-to-high-density lipoprotein cholesterol ratio. Group 1: low WHH + low WHR, Group 2: low WHH + high WHR, Group 3: high WHH + low WHR, Group 4: high WHH + high WHR. Model 1: crude. Model 2: adjusting for Age, Gender, Education, Ethnicity, Marital status, smoking, drinking. Model 3: adjusting for Age, Gender, Education, Ethnicity, Marital status, Smoking, Drinking, Albumin, AST, ALT, BUN, Scr, SUA, hsCRP.

### Analysis of Kaplan–Meier curves

**Figure 2:**
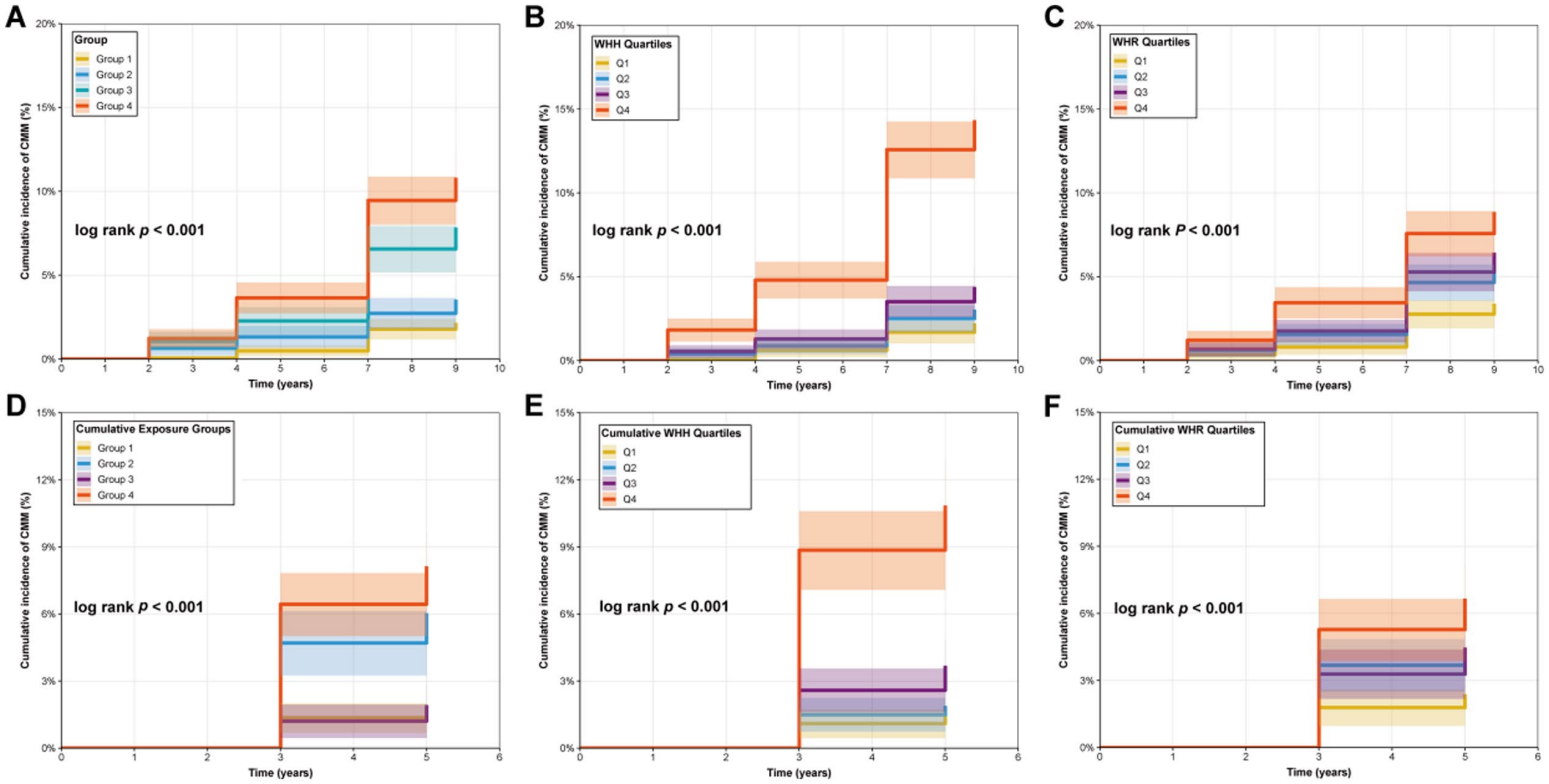
Kaplan–Meier curves for cumulative incidence of CMM. **(A)** By WHH-WHR combined groups; **(B)** By WHH quartiles; **(C)** By WHR quartiles; **(D)** By cuWHH-cuWHR combined groups; **(E)** By cuWHH quartiles; **(F)** By cuWHR quartiles. Log-rank *p* values are shown.

Kaplan–Meier curves demonstrated a clear separation between groups. Participants in Group 4 (high WHH + high WHR), the highest quartile (Q4) of both WHH and WHR, had the highest cumulative incidence of CMM, while those in Group 1 (low WHH + low WHR), the lowest quartile (Q1) of both biomarkers, showed the lowest cumulative incidence (log-rank *p* < 0.001, Figures 2A–C). Similar findings were observed across the cumulative exposure groups (Figures 2D–F).

### Nonlinear relationship between the WHH, WHR, and CMM risk

**Figure 3:**
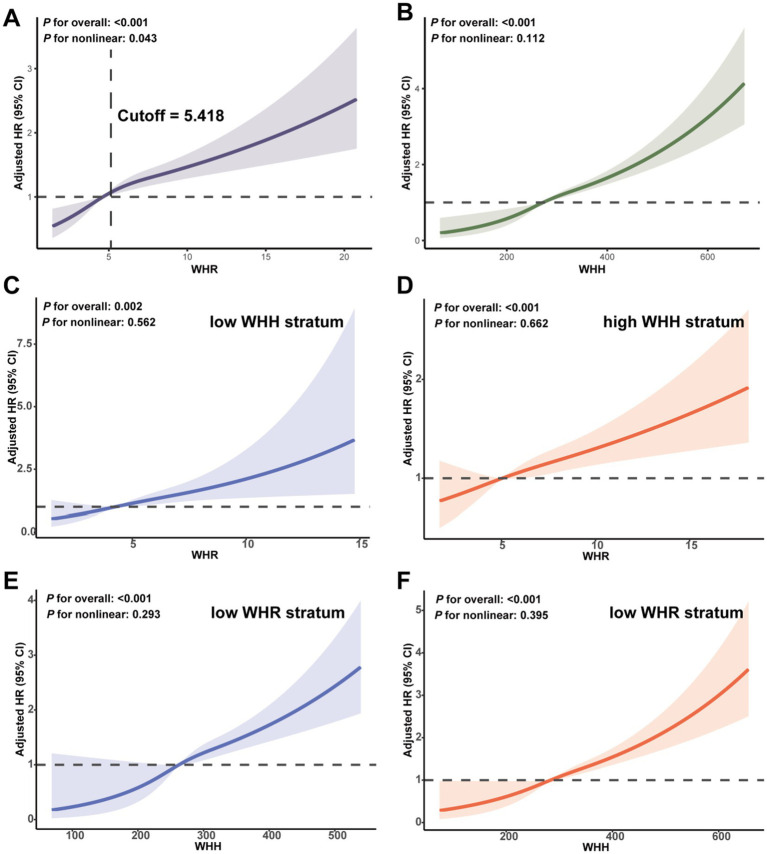
Restricted cubic spline (RCS) analysis of WHH, WHR and their stratified associations with CMM risk. **(A)** WHR and CMM risk; **(B)** WHH and CMM risk; **(C)** WHR RCS in low WHH group; **(D)** WHR RCS in high WHH group; **(E)** WHH RCS in low WHR group; **(F)** WHH RCS in high WHR group. Models were adjusted for all covariates.

To further explore the association of WHH and WHR with CMM risk, RCS analysis was performed. As shown in Figures 3A, a significant nonlinear relationship was observed between WHR and CMM risk (*p* for nonlinearity = 0.043). Accordingly, Cox piecewise regression was applied, and an optimal cutoff value of 5.418 was identified after full covariate adjustment (*p* for log-likelihood ratio test = 0.03998; Supplementary Table S9). Below this cutoff, each one-unit increase in WHR was associated with a 19.3% higher CMM risk, whereas above the cutoff, the corresponding increase was 5.2%. As illustrated in Figure 3B and Supplementary Figures S1A,B, WHH, cuWHR, and cuWHH exhibited linear associations with CMM risk (all *p* for overall < 0.05, *p* for nonlinearity > 0.05). Subsequent stratified RCS analyses, using the median as the threshold, revealed significant linear dose–response relationships between WHR and CMM risk in both low and high WHH strata (Figures 3C,D). Similarly, significant linear correlations between WHH and CMM risk were observed in both low and high WHR strata (Figures 3E,F). For cumulative exposure analyses, no significant association between cuWHR and CMM risk was detected in the low cuWHH stratum (*p* for overall > 0.05, *p* for nonlinearity > 0.05), whereas all other strata showed significant linear relationships (Supplementary Figures S1C–F). Consistent findings were also obtained when stratifying by the WHR cutoff of 5.418 (Supplementary Figure S2).

### Predictive value of WHH-WHR combined model on CMM

**Figure 4:**
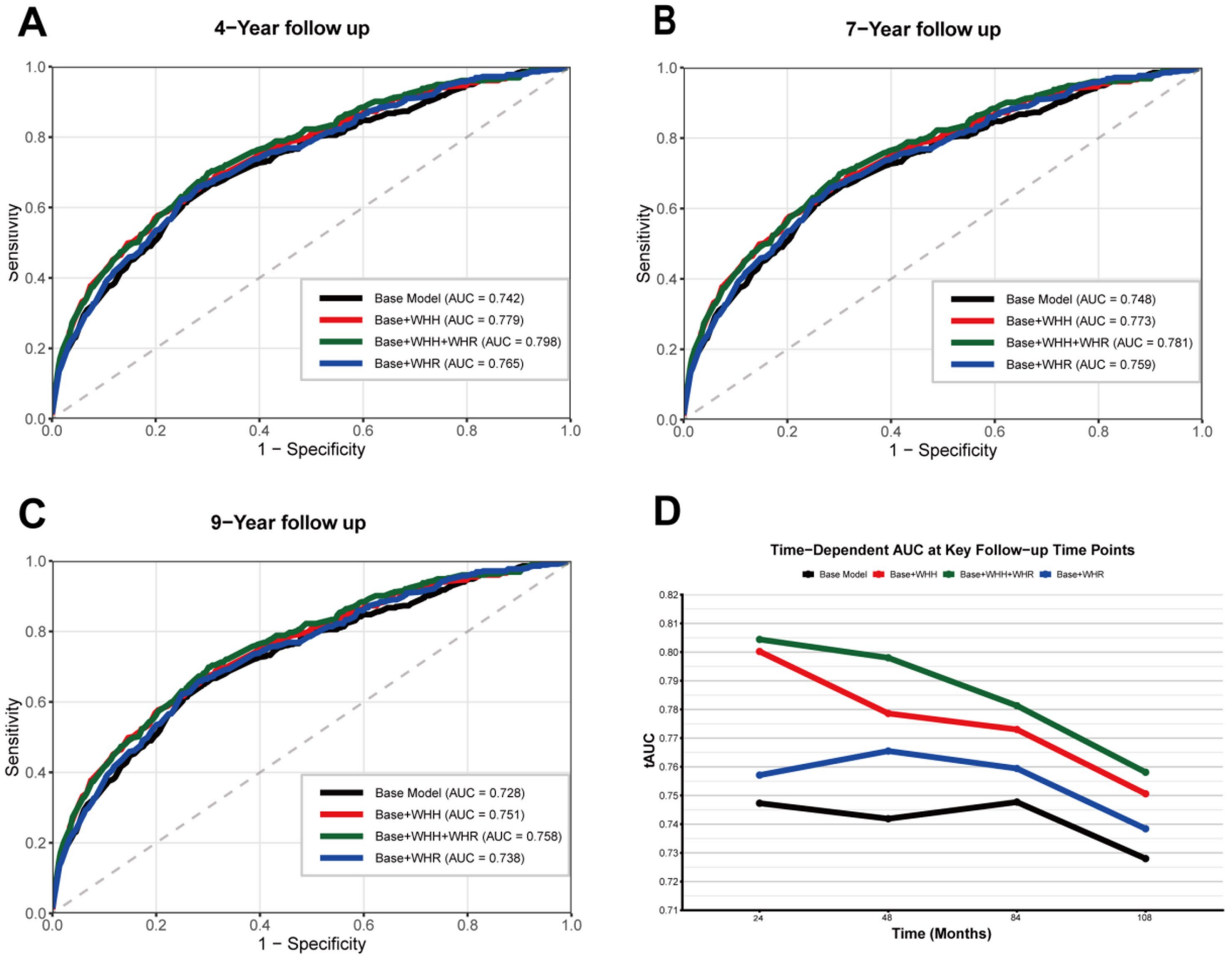
Predictive performance of WHH-WHR combined model for CMM risk. **(A-C)** Time-dependent ROC curves at 4, 7, and 9 years of follow-up; **(D)** Time-dependent AUC (tAUC) curves.

The predictive performance of each model for CMM risk was assessed using ROC curves, NRI, and IDI. The AUC of the WHH-WHR combined model was consistently higher than that of the base model and the individual WHH or WHR models at 4, 7, and 9 year follow-up (Figures 4A–C). Time-dependent AUC (tAUC) analysis further confirmed that the combined model outperformed the base model across all evaluation time points. The mean tAUC of the combined model was 0.7855 ± 0.0207, compared with 0.7412 ± 0.0088 for the base model, representing an average AUC improvement of approximately 0.042 (Figure 4D). The DeLong test for the 9-year cumulative incidence AUC showed that the WHH-WHR combined model had a significantly higher AUC than all other models (*p* < 0.05; Supplementary Table S10). NRI analysis revealed that adding WHH, WHR, or their combination to the base model significantly improved predictive performance, with AUC gains ranging from 0.007 to 0.056. The highest NRI was 0.4403 (95% CI: 0.1727–0.7417), indicating that the combined model achieved more accurate risk reclassification for 44.03% of participants relative to the base model. The highest IDI was 0.021 (95% CI: 0.011–0.033), further verifying the improved risk discrimination of the combined model. Collectively, these findings demonstrate that the WHH-WHR combined model provides significant incremental predictive value for CMM risk (Supplementary Table S11). Additionally, we further compared the WHH-WHR combined model with triglyceride-glucose-related indices (e.g., BMI, HbA1c, TyG-WHtR, CRP-TyG) and lipid-related indices (e.g., AIP, LCI). The WHH-WHR combined model yielded the highest AUC, demonstrating its robust and superior predictive performance (Supplementary Figure S3).

Similar results were also observed in the cumulative exposure models. The combination of cuWHH and cuWHR yielded the highest AUC of 0.7625. Compared with the base model, it significantly improved reclassification ability (NRI = 0.465, 95% CI: 0.271–0.650) and discrimination ability (IDI = 0.028, 95% CI: 0.011–0.055; Supplementary Figure S4; Supplementary Table S12). Consistently, the combined assessment of WHH and WHR also showed the high AUC in the cross-sectional study (Supplementary Figure S5).

### Mediation analyses

**Figure 5:**
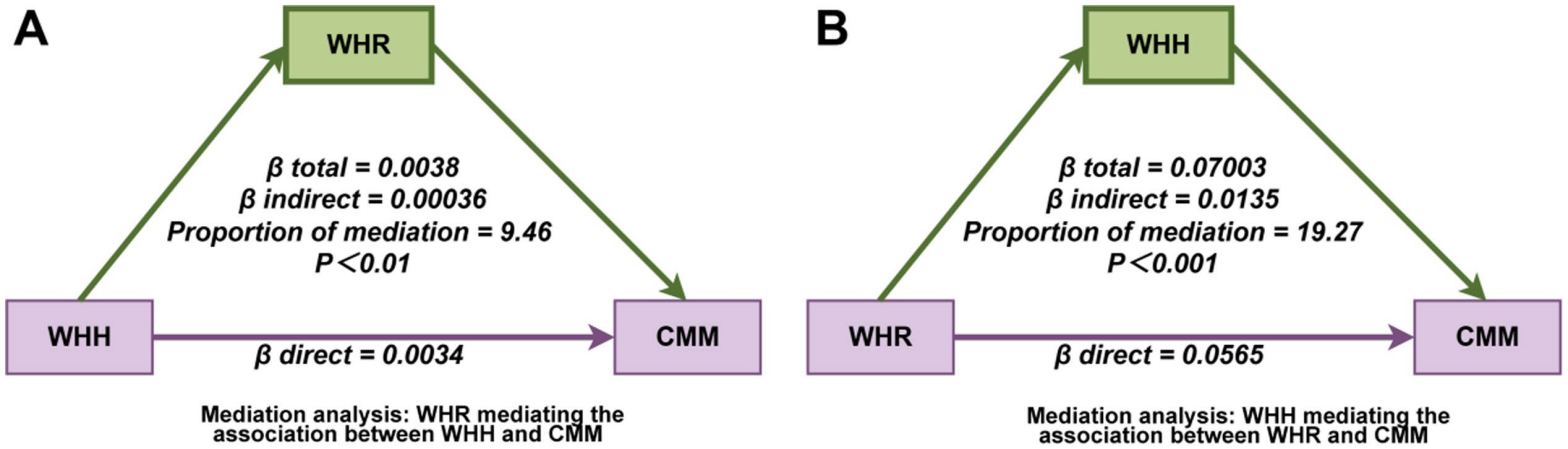
Bidirectional mediation effects between WHH and WHR on CMM risk, the proportions mediated were calculated after adjusting for all covariates.

A bidirectional mediation analysis was conducted to further elucidate the interrelationships between WHH, WHR, and CMM risk (Figure 5). Following full covariate adjustment, WHR significantly mediated 9.46% of the association between WHH and CMM risk, whereas WHH significantly mediated 19.27% of the association between WHR and CMM risk. WHH exerted a more pronounced indirect contribution to WHR-associated risk, suggesting that WHH may reflect an earlier stage of metabolic dysregulation, and its abnormalities may elevate CMM risk by altering the inflammatory-lipid profile.

### Additive and multiplicative interaction analyses and WQS regression

Interaction analyses were conducted to assess the combined effects of baseline and cumulative WHH and WHR (Supplementary Tables S13, S14). In the fully adjusted models, the SI, AP, RERI, and multiplicative interaction were all non-significant, indicating that WHH and WHR may independently affect CMM risk without synergistic or antagonistic interactions. WQS regression was performed to evaluate the combined contribution of individual components of WHH and WHR to CMM risk (Supplementary Figure S6). The mixture exposure was significantly and positively associated with CMM risk (OR = 2.10, 95% CI: 1.50–2.94, *p* < 0.001). The contribution weights of each component were as follows: WC (40.9%), HbA1c (39.4%), WBC (11.4%), height (7.3%), and HDL-C (1.1%).

### Subgroup analysis

**Figure 6:**
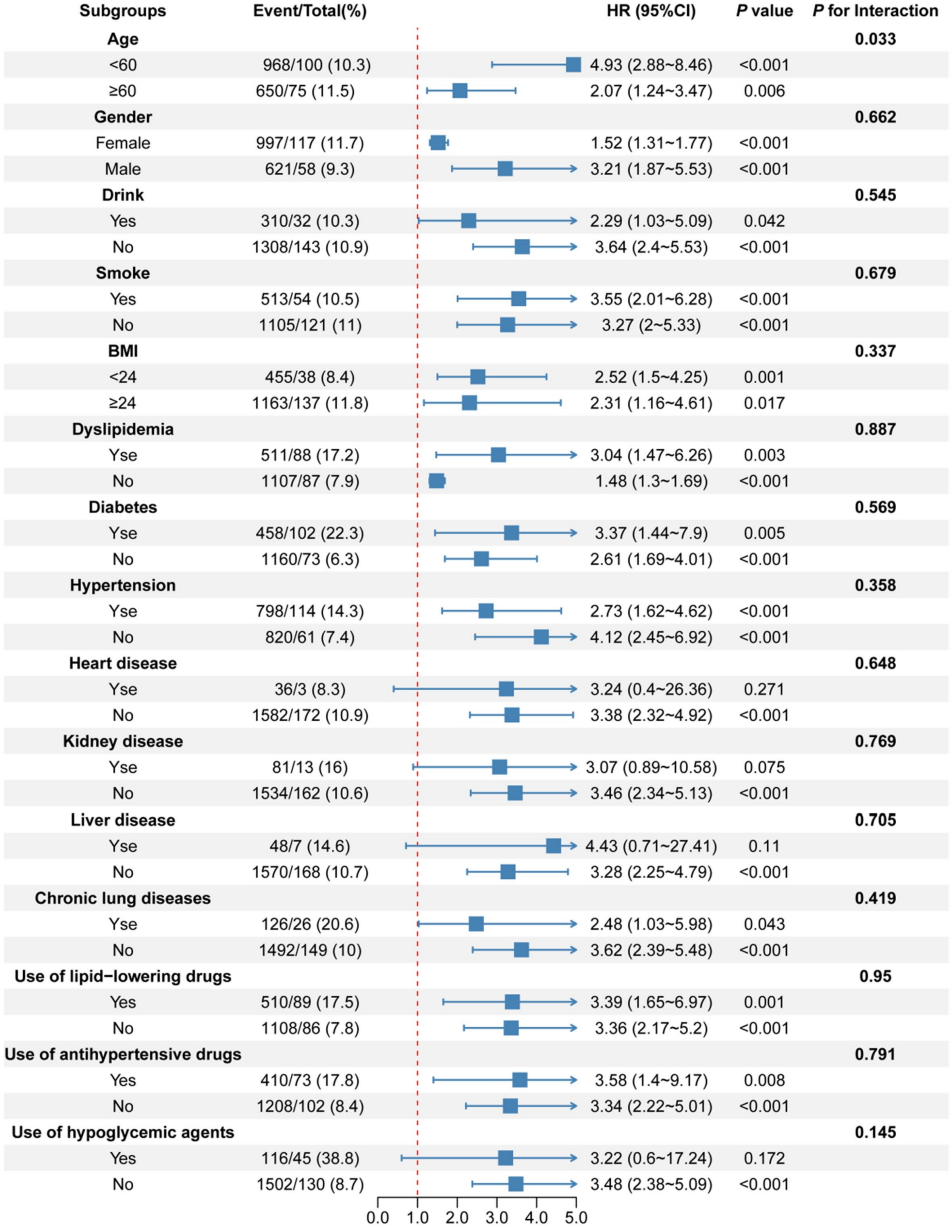
Subgroup analysis for the association between high WHH + high WHR group and CMM risk.

Subgroup analyses were performed to explore the association between the high WHH + high WHR group and CMM risk, stratified by age (<60 vs. ≥60 years), sex, alcohol consumption, smoking, BMI (<24 vs. ≥24 kg/m^2^), dyslipidemia, diabetes, hypertension, heart disease, kidney disease, liver disease, chronic lung disease, and use of lipi-lowering, antihypertensive, and hypoglycemic medications (Figure 6). After full covariate adjustment, a significant interaction was detected between age group and CMM risk, with participants aged <60 years exhibiting a higher risk (*p* for interaction = 0.033). No significant interactions were observed for other variables (all *p* for interaction > 0.05). Similarly, no significant interactions were found for the high cuWHH + high cuWHR group across any stratification (*p* for interaction > 0.05) (Supplementary Figure S7). These findings indicate favorable predictive performance across diverse population subgroups.

### Sensitivity analysis

To further validate the robustness of our findings, a series of sensitivity analyses were performed. First, analyses were repeated using non-imputed data to assess the influence of multiple imputation. Second, participants with baseline diabetes, heart disease, or stroke were excluded to eliminate confounding by pre-existing conditions. Third, participants receiving treatment for diabetes, dyslipidemia, or hypertension at baseline were excluded to control for medication-related confounding. Fourth, individuals who developed CMM within the first 2 years of follow-up were excluded to minimize reverse causation bias. Fifth, participants with baseline hypertension were excluded to evaluate its potential confounding effect. Sixth, participants with baseline dyslipidemia were excluded to rule out its interference with the observed association. Finally, extreme values (the lowest and highest 1%) of baseline and cumulative WHH and WHR were removed to assess the impact of outliers (Supplementary Tables S15–S28).

## Discussion

In this 9-year nationwide cohort study, we systematically evaluated the association of the composite biomarkers WHR and WHH, as well as their combination, with CMM risk. Our results demonstrated that both baseline and cumulative exposure to WHH and WHR were significantly positively associated with elevated CMM risk, and their combination enabled more effective identification of high-risk individuals. The combined model exhibited better predictive performance for CMM than the base model, individual biomarkers, and widely used indices including AIP and CRP-TyG, and it also achieved substantial improvements in risk reclassification. WHH accounted for a larger contribution weight and exerted an indirect effect in the associated pathway, suggesting that it may reflect an earlier stage of metabolic dysregulation. To further verify the reliability of our conclusions, we additionally performed external validation using cross-sectional data from the NHANES dataset. Findings from this external population also supported the value of combined WHH and WHR in identifying individuals at high risk of CMM.

Existing studies have verified that WHR is a strong predictor of heart disease, while WHH can effectively predict diabetes (11–13). This study confirmed that both WHH and WHR were independently associated with CMM risk, regardless of whether baseline single measurements or cumulative exposure levels were used. This finding is well aligned with their underlying pathophysiological mechanisms. As a composite index combining waist-to-height ratio and long-term glycemic status, WHH directly reflects visceral obesity and impaired glucose metabolism, two core drivers of CMM (31, 32). WHtR is a reliable marker for assessing central obesity and visceral fat accumulation. Studies have demonstrated that it is highly prevalent among patients with heart failure and strongly linked to adverse clinical events. Compared with WC and waist-to-hip ratio, WHtR can more accurately reflect central obesity (33, 34). When subcutaneous fat storage reaches its maximum capacity, excess fat accumulates in visceral organs including the pancreas, skeletal muscle and liver. Enlarged visceral adipocytes release substantial free fatty acids (FFAs) and trigger cellular hypoxia, stress and apoptosis, while recruiting immune cells to sustain a pro-inflammatory state. In turn, inflammation and FFAs damage pancreatic *β*-cells, impair GLUT4-mediated glucose transport and uptake in skeletal muscle, and promote insulin resistance. They also stimulate aldosterone secretion, which causes elevated blood pressure, myocardial fibrosis and ventricular dysfunction, ultimately resulting in adverse clinical outcomes (35–38). HbA1c is the gold standard for assessing long-term glycemic control. Elevated HbA1c indicates sustained hyperglycemia, which readily promotes the formation of advanced glycation end products (AGEs). AGEs further trigger oxidative stress, endothelial dysfunction, atherosclerosis and other pathological changes (39–41). WHR reflects systemic inflammatory burden and HDL-C levels. Chronic inflammation impairs insulin sensitivity and damages pancreatic β-cells. Via multiple signaling pathways, it also affects the formation and stability of atherosclerotic plaques, thereby increasing the risk of heart disease, stroke and diabetes (42, 43). Reduced HDL-C levels indicate diminished anti-atherosclerotic capacity (44). Accordingly, an elevated WHR indicates an imbalance between pro-inflammatory activity and anti-atherosclerotic protection. Our results revealed that the combined assessment of WHH and WHR was significantly positively correlated with CMM risk. Participants in Group 4 exhibited a threefold higher disease risk compared with those in Group 1. Analyses of AUC, NRI and IDI collectively validated the favorable predictive performance of this combined model. This indicates that their joint use can more comprehensively capture the multidimensional pathological features of CMM. The novelty of this study lies in combining two composite indices that reflect obesity, metabolism, inflammation and lipid profiles, and verifying their predictive value. CMM progression is driven by multiple intertwined mechanisms rather than a single pathway, following a complex cascade: visceral obesity characterized by aberrant fat distribution in the liver, pancreas and skeletal muscle → polarization of M1-like ATMs → release of chemokines and cytokines → pancreatic *β*-cell apoptosis, insulin resistance and dyslipidemia (45–48). Visceral obesity often acts as the initial trigger of this pathological cascade, mediating vascular alterations via crosstalk among immune, hepatic, lymphatic, muscular and adipose tissues (49, 50). Results from mediation analysis and WQS regression suggest that central obesity represents an early and core link in the pathogenesis of CMM. Targeting visceral obesity-related metabolic abnormalities may help reduce the risk of CMM.

In addition, we compared the combined model with established metabolic and inflammatory markers. The WHH-WHR model yielded the highest AUC value. ROC analyses based on external NHANES validation data further confirmed that this combined model maintained superior predictive performance in an independent population, demonstrating its good stability. These findings support the clinical application of the WHH-WHR combination for CMM risk stratification, as it is simple to calculate and cost-effective.

This study identified a non-linear dose–response relationship between WHR and CMM risk, which may be attributed to the dual biological effects of HDL-C. Traditionally, HDL-C is recognized to exert anti-inflammatory, antioxidant and reverse cholesterol transport functions. Nevertheless, emerging evidence indicates that HDL-C may undergo functional alterations under certain internal conditions, leading to cholesterol deposition and pro-inflammatory effects. HDL-C exhibits a U-shaped association with diabetes, CVD and all-cause mortality, and its optimal concentration range differs across various diseases (51, 52). Despite this non-linear association, our results demonstrate that elevated WHR correlates with increased CMM risk. This highlights the need for regular WHR monitoring in clinical settings.

Subgroup analyses indicated that participants under 60 years of age in Group 4 (high WHH + high WHR) had a higher risk of CMM. This is probably because metabolic disorders in younger people are still at an early and intervenable stage, and central obesity as well as inflammatory burden exert a more pronounced effect on the development of CMM. Among those aged over 60, age itself becomes the leading risk factor, which impairs the ability of metabolic indicators for risk stratification to a certain degree.

All sensitivity analyses yielded results consistent with the main findings, further confirming the stability and reliability of the study conclusions.

This study has several strengths. Firstly, the primary analysis relied on a large nationally representative prospective cohort. We further validated our results externally in an independent large representative cross-sectional dataset covering different populations. This dual-dataset design confirms that our conclusions are applicable across diverse populations. Secondly, we integrated WHH and WHR to predict CMM risk. Given the complex pathogenic mechanisms of CMM, this combination enables a more comprehensive evaluation. Thirdly, we performed comprehensive mediation analysis, interaction analysis, WQS regression, subgroup analysis and sensitivity analysis. These analyses explored the underlying mechanistic links and population-specific effects, and further validated the robustness of our findings. Finally, considering the dynamic progression of cardiometabolic diseases, the two-time-point cumulative exposure indicators used in this study can more accurately reflect disease risk.

This study also has several limitations. First, although a comprehensive set of covariates was adjusted for, potential residual confounding may still exist. Secondly, CMM diagnosis was primarily based on self-reported physician diagnoses and laboratory measurements, which may lead to recall bias and disease misclassification and thereby affect the accuracy of the results. Thirdly, this study was based on data from CHARLS and NHANES. Although multiple approaches were used to verify the robustness of the findings, the generalizability of the prediction model remains to be further validated in future research. Fourthly, due to data availability, cumulative exposure was only assessed at two time points, which fails to capture long-term dynamic trends. Further analyses with multi-timepoint data will be conducted to explore the association between trajectory changes and disease risk. Finally, several subgroups such as patients with liver and kidney diseases had relatively small sample sizes, which may compromise the stability of corresponding subgroup findings. Additional validation with larger cohorts is required in future studies.

## Conclusion

This study shows that elevated WHH and WHR are jointly associated with an increased risk of CMM. The combination of WHH and WHR exhibits favorable diagnostic performance, which is superior to conventional metabolic risk indicators and BMI. These findings indicate that they serve as a simple and effective biomarker panel for primary prevention. Early monitoring and combined assessment help identify individuals at high risk.

## Data Availability

The datasets presented in this study can be found in online repositories. The names of the repository/repositories and accession number(s) can be found below: http://charls.pku.edu.cn/, https://www.cdc.gov/nchs/nhanes/.

## Footnotes

1
www.cdc.gov/nchs/nhanes

## Glossary

AIP: Atherogenic index of plasma
AUC: Area under the curve
BMI: Body mass index
CHARLS: China Health and Retirement Longitudinal Study
CI: Confidence interval
CMM: Cardiometabolic multimorbidity
CRP: C-reactive protein
cuWHH: Cumulative waist-to-height-hemoglobin A1c
cuWHR: Cumulative white blood cell-to-HDL cholesterol ratio
HDL-C: high-density lipoprotein cholesterol
HbA1c: Hemoglobin A1c
HR: Hazard ratio
IDI: Integrated discrimination improvement
IR: Insulin resistance
MICE: Multiple imputation by chained equations
NHANES: National Health and Nutrition Examination Survey
NRI: Net reclassification improvement
OR: Odds ratio
RCS: Restricted cubic spline
ROC: Receiver operating characteristic curve
TyG: Triglyceride-glucose index
WC: Waist circumference
WBC: White blood cell
WHR: White blood cell-to-HDL cholesterol ratio
WHH: Waist-to-height-hemoglobin A1c
WHtR: Waist-to-height ratio
WQS: Weighted quantile sum

## References

[1] Glynn LG. Multimorbidity: another key issue for cardiovascular medicine. Lancet. (2009) 374:1421–2. doi: 10.1016/S0140-6736(09)61863-8, 19854371

[2] Angelantonio ED Kaptoge S Wormser D Willeit P Butterworth AS Bansal N . Association of cardiometabolic multimorbidity with mortality. JAMA. (2015) 314:52–60. doi: 10.1001/jama.2015.7008, 26151266 PMC4664176

[3] Yang L Zhang Z Zhang J Miao J Zhang H Du Y . Stage-specific risk factors of cardiometabolic multimorbidity: a systematic review and meta-analysis from incidence to mortality. Ageing Res Rev. (2026) 114:102991. doi: 10.1016/j.arr.2025.102991 41390099

[4] Kunutsor SK Young JS Laukkanen JA. Visceral adiposity index is associated with cardiometabolic multimorbidity and improves risk prediction: the English longitudinal study of ageing. Mayo Clin Proc. (2026) 101:259–69. doi: 10.1016/j.mayocp.2025.09.021, 41467869

[5] Aychiluhm Setognal B Ross Allen G Vivian I Subash T Ahmed Kedir Y. Central obesity and its association with youth physical and mental health: evidence from the Australian National Health Survey. BMC Med. (2025) 23:709. doi: 10.1186/s12916-025-04538-5, 41299659 PMC12752446

[6] Cai X Liao Y Yang X Liang Y Ma J Liu R . Body roundness index associated with cardiometabolic multimorbidity and mortality: a multistate model. Obesity (Silver Spring). (2025) 33:2377–86. doi: 10.1002/oby.70032, 41017239 PMC12636056

[7] Dong H Chang L Tian T Shi R Yu K Wang C . Mechanism-guided pharmacotherapy for cardiometabolic multimorbidity: from pathophysiology to phenotype-prioritized treatment. Front Endocrinol (Lausanne). (2025) 16:1724965. doi: 10.3389/fendo.2025.1724965, 41404511 PMC12702714

[8] Lin Y Tao J Wang H Guan H Liu X Dong X . The association of C-reactive protein-triglyceride-glucose index with cardiometabolic multimorbidity in middle-aged and older adults: evidence from two cohort studies. Cardiovasc Diabetol. (2026) 25:90. doi: 10.1186/s12933-026-03109-z 41688996 PMC13005544

[9] Chenyang L Xiaoqin L Yifan C Jiafeng L Shengyuan G. Predictive value of an integrated insulin resistance and lipometabolic score for cardiometabolic multimorbidity in older adults: a UK cohort study. Cardiovasc Diabetol. (2026) 25:3064. doi: 10.1186/s12933-025-03064-1, 41639900 PMC12947332

[10] Jin T Tang X Han Y Fan H Qin Q Jiang H . Relationship between nine triglyceride-glucose-related indices and cardiometabolic multimorbidity incidence in patients with cardiovascular-kidney-metabolic syndrome stage 0-3: a nationwide prospective cohort study. Cardiovasc Diabetol. (2026) 25:36. doi: 10.1186/s12933-026-03077-4, 41526923 PMC12879366

[11] Hu W Feng H Xu X Sun Z Lu C Liu Y . CABIT: a novel biomarkers-integrated inflammatory risk tool for ischemic heart disease developed in the USA and prospectively validated in China. J Transl Med. (2026) 24:249. doi: 10.1186/s12967-026-07712-2, 41572286 PMC12910730

[12] Wu T Zheng Y Xiu W Wu T-T Zheng Y-Y Xiu W-J . White blood cell counts to high-density lipoprotein cholesterol ratio, as a novel predictor of long-term adverse outcomes in patients after percutaneous coronary intervention: a retrospective cohort study. Front Cardiovasc Med. (2021) 8:616896. doi: 10.3389/fcvm.2021.616896, 34307487 PMC8295559

[13] Wang L Wang W Ji Y Zong G. The product of waist-to-height ratio and glycated hemoglobin, a novel predictor of diabetes in east Asian populations: insights from two large east Asian cohort studies. Diabetol Metab Syndr. (2025) 17:274. doi: 10.1186/s13098-025-01859-6, 40671120 PMC12269157

[14] Rheinheimer J de Souza BM Cardoso NS Bauer AC Crispim D. Current role of the NLRP3 inflammasome on obesity and insulin resistance: a systematic review. Metabolism. (2017) 74:1–9. doi: 10.1016/j.metabol.2017.06.002, 28764843

[15] Hong N Lin Y Ye Z Yang C Huang Y Duan Q . The relationship between dyslipidemia and inflammation among adults in east coast China: a cross-sectional study. Front Immunol. (2022) 13:937201. doi: 10.3389/fimmu.2022.937201, 36032093 PMC9403313

[16] Herbert T Gianluca I Antonio G Adolph Timon E. Adipokines: masterminds of metabolic inflammation. Nat Rev Immunol. (2025) 25:250–65. doi: 10.1038/s41577-024-01103-8, 39511425

[17] De Heredia FP Gómez-Martínez S Marcos A. Obesity, inflammation and the immune system. Proc Nutr Soc. (2012) 71:332–8. doi: 10.1017/S0029665112000092, 22429824

[18] Schleh MW Caslin HL Garcia JN Mashayekhi M Srivastava G Bradley AB . Metaflammation in obesity and its therapeutic targeting. Sci Transl Med. (2023) 15:eadf9382. doi: 10.1126/scitranslmed.adf9382, 37992150 PMC10847980

[19] Zhao Y Hu Y Smith JP Strauss J Yang G. Cohort profile: the China health and retirement longitudinal study (CHARLS). Int J Epidemiol. (2014) 43:61–8. doi: 10.1093/ije/dys203, 23243115 PMC3937970

[20] Ma X Ma X Wang Y Qiu G Zhang C. Associations of cumulative exposure and dynamic trajectories of the C-reactive protein-triglyceride-glucose index with incident cardiovascular disease in middle-aged and older Chinese adults: a nationwide cohort study. Cardiovasc Diabetol. (2025) 24:303. doi: 10.1186/s12933-025-02869-4, 40713770 PMC12296596

[21] Zheng W Man Z Ren Y Li Y Zhu X Wang L . Association of the triglyceride glucose-Chinese visceral adiposity index with incident cardiometabolic multimorbidity in middle-aged and older adults: a nationwide prospective cohort study. Cardiovasc Diabetol. (2026) 25:3091. doi: 10.1186/s12933-026-03091-6, 41639722 PMC12964883

[22] Zhao X Xu X Yan Y Lipnicki DM Pang T Crawford JD . Independent and joint associations of cardiometabolic multimorbidity and depression on cognitive function: findings from multi-regional cohorts and generalisation from community to clinic. Lancet Reg Health West Pac. (2024) 51:101198. doi: 10.1016/j.lanwpc.2024.101198, 39308753 PMC11416683

[23] Tai XY Veldsman M Lyall DM Littlejohns TJ Langa KM Husain M . Cardiometabolic multimorbidity, genetic risk, and dementia: a prospective cohort study. Lancet Healthy Longev. (2022) 3:e428–36. doi: 10.1016/S2666-7568(22)00117-9, 35711612 PMC9184258

[24] Huang M Fu R Zhao X Liu T Li X Jiang W. Life's essential 8 and progression of cardiometabolic multimorbidity trajectory: a prospective study of UK biobank. Eur J Prev Cardiol. (2025). doi: 10.1093/eurjpc/zwaf247, 40285703

[25] Yu J Yi Q Chen G Hou L Liu Q Xu Y . The visceral adiposity index and risk of type 2 diabetes mellitus in China: a national cohort analysis. Diabetes Metab Res Rev. (2022) 38:e3507. doi: 10.1002/dmrr.3507, 34679251

[26] Li W Shen C Kong W Zhou X Fan H Zhang Y . Association between the triglyceride glucose-body mass index and future cardiovascular disease risk in a population with cardiovascular-kidney-metabolic syndrome stage 0-3: a nationwide prospective cohort study. Cardiovasc Diabetol. (2024) 23:292. doi: 10.1186/s12933-024-02352-6, 39113004 PMC11308445

[27] Yuting Z Yuan Z Yuqing H Yane Y Qing Z Du Y. Association of cumulative cholesterol-HDL-glucose index with blood pressure changes and risk of new-onset hypertension in middle-aged and older adults: a cohort study. Cardiovasc Diabetol. (2026) 25:49. doi: 10.1186/s12933-026-03081-8, 41559691 PMC12903659

[28] Lai H Deng C Liao C Tian R Liu K Luo Z . Joint assessment of insulin resistance surrogate indices and basal metabolic rate for primary prevention of cardiometabolic multimorbidity: evidence from the China health and retirement longitudinal study (2011-2020). Cardiovasc Diabetol. (2011) 25:37. doi: 10.1186/s12933-025-03067-y, 41527106 PMC12888234

[29] Williams B Mancia G Spiering W Agabiti Rosei E Azizi M Burnier M . 2018 ESC/ESH guidelines for the management of arterial hypertension. Eur Heart J. (2018) 39:3021–104. doi: 10.1093/eurheartj/ehy339, 30165516

[30] Wang C He S Xie G Zhang S Xiong Z Lu H . Associations of longitudinal trajectories of triglyceride-glucose index combined with classical and novel obesity indices and cardiovascular disease: evidence from a nationwide prospective cohort study in China. Cardiovasc Diabetol. (2025) 24:431. doi: 10.1186/s12933-025-02972-6, 41225604 PMC12613409

[31] Wan S Shi M Gao Y. Joint associations of lung function of both general and abdominal obesity with cardiometabolic multimorbidity: a cross-sectional study. Front Med (Lausanne). (2026) 13:1761219. doi: 10.3389/fmed.2026.1761219, 41884126 PMC13008965

[32] Zhao-Xuan L Bing-Qing D Liang C Heng-Le W Hong Z. Data-driven glycolipid network phenotypes reveal a graded risk spectrum for cardiometabolic multimorbidity: a prospective study in middle-aged and older Chinese adults. Lipids Health Dis. (2026) 25:80. doi: 10.1186/s12944-026-02894-6, 41673664 PMC12998046

[33] Peikert A Vaduganathan M Claggett BL Kulac IJ Litwin S Zile M . Near-universal prevalence of central adiposity in heart failure with preserved ejection fraction: the PARAGON-HF trial. Eur Heart J. (2025) 46:2372–90. doi: 10.1093/eurheartj/ehaf057, 39873282 PMC12208775

[34] Feng Q Bešević J Conroy M Omiyale W Woodward M Lacey B . Waist-to-height ratio and body fat percentage as risk factors for ischemic cardiovascular disease: a prospective cohort study from UK biobank. Am J Clin Nutr. (2024) 119:1386–96. doi: 10.1016/j.ajcnut.2024.03.018, 38839194 PMC11196863

[35] Packer M. The epicardial adipose inflammatory triad: coronary atherosclerosis, atrial fibrillation, and heart failure with a preserved ejection fraction. Eur J Heart Fail. (2018) 20:1567–9. doi: 10.1002/ejhf.1294, 30225884

[36] Naryzhnaya NV Koshelskaya OA Kologrivova IV Kharitonova OA Evtushenko VV Boshchenko AA. Hypertrophy and insulin resistance of epicardial adipose tissue adipocytes: association with the coronary artery disease severity. Biomedicine. (2021) 9:64. doi: 10.3390/biomedicines9010064, 33440802 PMC7827040

[37] Packer M. Leptin-aldosterone-neprilysin Axis: identification of its distinctive role in the pathogenesis of the three phenotypes of heart failure in people with obesity. Circulation. (2018) 137:1614–31. doi: 10.1161/CIRCULATIONAHA.117.032474, 29632154

[38] Khanna D Khanna S Khanna P Kahar P Patel BM. Obesity: a chronic low-grade inflammation and its markers. Cureus. (2022) 14:e22711. doi: 10.7759/cureus.22711, 35386146 PMC8967417

[39] Hirai T Fujiyoshi K Yamada S Matsumoto T Kikuchi J Ishida K . Advanced glycation end products are associated with diabetes status and physical functions in patients with cardiovascular disease. Nutrients. (2022) 14:32. doi: 10.3390/nu14153032, 35893886 PMC9330730

[40] Eskesen K Jensen MT Galatius S Vestergaard H Hildebrandt P Marott JL . Glycated haemoglobin and the risk of cardiovascular disease, diabetes and all-cause mortality in the Copenhagen City heart study. J Intern Med. (2013) 273:94–101. doi: 10.1111/j.1365-2796.2012.02594.x, 23009556

[41] Evans PC Vilahur G Kleinbongard P Osto E Remme CA Madonna R . Novel cardiovascular metabolic risk factor mechanisms and therapeutic opportunities. Eur Heart J. (2026) 6:116. doi: 10.1093/eurheartj/ehag116, 41790457

[42] She X Yang J Ren B Yang X Cheng X Tian T . Associations between inflammatory indices derived from complete blood counts and mortality risk in diabetes and prediabetes patients: a cohort study from NHANES 1999--2018. Diabetes Res Clin Pract. (1999) 232:113112. doi: 10.1016/j.diabres.2026.113112, 41577297

[43] Welsh C Welsh P Mark PB Celis-Morales CA Lewsey J Gray SR . Association of Total and Differential Leukocyte Counts with Cardiovascular Disease and mortality in the UK biobank. Arterioscler Thromb Vasc Biol. (2018) 38:1415–23. doi: 10.1161/ATVBAHA.118.310945, 29699973

[44] Li P Cai J Yuan S Li Y Shi H Liang C . The relationship between triglyceride to high-density lipoprotein cholesterol ratio and cardiovascular high risk: a cross-sectional investigation. Front Endocrinol (Lausanne). (2025) 16:1688624. doi: 10.3389/fendo.2025.1688624, 41393287 PMC12695605

[45] Lu X Kong X Wu H Hao J Li S Gu Z . UBE2M-mediated neddylation of TRIM21 regulates obesity-induced inflammation and metabolic disorders. Cell Metab. (2023) 35:1390–1405.e8. doi: 10.1016/j.cmet.2023.05.011, 37343564

[46] Rohm TV Meier DT Olefsky JM Donath MY. Inflammation in obesity, diabetes, and related disorders. Immunity. (2022) 55:31–55. doi: 10.1016/j.immuni.2021.12.013, 35021057 PMC8773457

[47] Li J Li Y Zhou X Yang S Liu D Wen H . Adipocytes orchestrate obesity-related chronic inflammation through β2-microglobulin. Signal Transduct Target Ther. (2025) 10:394. doi: 10.1038/s41392-025-02486-3, 41330906 PMC12672573

[48] Xie C Yuan Y Wang Y Qi C Wang W An C . Beyond discrete diagnoses: conceptualizing obesity-associated metabolic disorders as a unified, dynamic continuum. Curr Obes Rep. (2025) 14:81. doi: 10.1007/s13679-025-00673-5, 41261312

[49] Liu J Yang D. Current knowledge of obesity-related vascular injury. Obes Rev. (2025) 2025:e70080. doi: 10.1111/obr.70080 41436089

[50] Donath MY Drucker DJ. Obesity, diabetes, and inflammation: pathophysiology and clinical implications. Immunity. (2025) 58:2373–82. doi: 10.1016/j.immuni.2025.09.011, 41045922

[51] Lu J Han G Liu X Chen B Peng K Shi Y . Association of high-density lipoprotein cholesterol with all-cause and cause-specific mortality in a Chinese population of 3.3 million adults: a prospective cohort study. Lancet Reg Health West Pac. (2024) 42:100874. doi: 10.1016/j.lanwpc.2023.100874, 38357392 PMC10865023

[52] Lui DTW Li L Liu X Xiong X Tang EHM Lee CH . The association of HDL-cholesterol levels with incident major adverse cardiovascular events and mortality in 0.6 million individuals with type 2 diabetes: a population-based retrospective cohort study. BMC Med. (2024) 22:586. doi: 10.1186/s12916-024-03810-4, 39696353 PMC11657474

