# Supplementary material for: Joint assessment of white blood cell-to-HDL cholesterol ratio and waist-to-height-hemoglobin A1c for cardiometabolic multimorbidity risk: a prospective cohort study and cross-sectional study
Source: Front Nutr. 2026 Jun 30;13:1855163. doi: 10.3389/fnut.2026.1855163 (PMC13364917; doi:10.3389/fnut.2026.1855163)
Supplement: Supplementary file 1 [file Data_Sheet_1.PDF]

Table S1 Data missing situation in the original cohort

Variable	Missing Count	Missing Percent
Cystatinc, mg/dL	1540	26.03
Drinking	1516	25.63
SBP, mmHg	53	0.90
DBP, mmHg	53	0.90
Liver disease	25	0.42
Weight, kg	20	0.34
BMI, kg/m^2	20	0.34
Kidney disease	17	0.29
Depression	19	0.32
LDL-C, mg/dL	13	0.22
Chronic lung diseases	10	0.17
TC, mg/dL	8	0.14
TG, mg/dL	6	0.1
Gender	4	0.07
UA, mg/dL	1	0.02
Hb, g/dL	2	0.03
BUN, mg/dL	1	0.02
CRP, mg/dL	1	0.02

SBP, systolic blood pressure; DBP, diastolic blood pressure; BMI, body mass index; LDL-C, low-density lipoprotein cholesterol; TC, total cholesterol; TG, triglycerides; UA, Serum uric acid; Hb, Hemoglobin; BUN, Blood Urea Nitrogen; CRP, c-reactive protein.

Table S2 Data missing situation of cumulative exposure cohort

Variable	Missing Count	Missing Percent
Cystatinc, mg/dL	1060	26.36
Drinking	1021	25.39
SBP, mmHg	32	0.8
DBP, mmHg	31	0.77
Liver disease	14	0.35
Kidney disease	14	0.35
Depression	13	0.32
BMI, kg/m2	12	0.3
LDL-C, mg/dL	10	0.25
Scr, mg/dL	10	0.25
Chronic lung diseases	9	0.22
TC, mg/dL	6	0.15
Gender	4	0.1
TG, mg/dL	4	0.1
UA, mg/dL	1	0.02
BUN, mg/dL	1	0.02
CRP, mg/dL	1	0.02

Hb, g/dL	1	0.02

SBP, systolic blood pressure; DBP, diastolic blood pressure; BMI, body mass index; LDL-C, low-density lipoprotein cholesterol; TC, total cholesterol; TG, triglycerides; UA, Serum uric acid; Hb, Hemoglobin; BUN, Blood Urea Nitrogen; CRP, c-reactive protein.

Table S3 Multicollinearity analysis in the association analysis between WHH-WHR combined groups and CMM

Variables	GVIF	Df	GVIF^(1/(2*Df))
WHH-WHR combined group	1.43	1	1.196
Age	1.704	1	1.305
Gender	2.384	1	1.544
Marital status	1.192	3	1.03
Education	1.357	2	1.079
Smoking	1.688	1	1.299
Drinking	1.322	1	1.15
SBP, mmHg	3.335	1	1.826
DBP, mmHg	2.454	1	1.566
UA, mg/dL	1.719	1	1.311
Scr, mg/dL	2.161	1	1.47
BUN, mg/dL	1.311	1	1.145
CRP, mg/dL	1.456	1	1.207
Cystatinc, mg/dL	1.728	1	1.314
Hb, g/dL	1.215	1	1.102
Hypertension	3.406	1	1.846
Chronic lung diseases	1.076	1	1.037
Liver disease	1.055	1	1.027
Kidney disease	1.129	1	1.063
Depression	1.022	1	1.011
Use of antihypertensive drugs	3.264	1	1.807
Use of hypoglycemic agents	1.545	1	1.243

GVIF, generalized variance inflation factor; Df, degrees of freedom; SBP, systolic blood pressure; DBP, diastolic blood pressure; UA, Serum uric acid; Scr, Serum Creatinine; BUN, Blood Urea Nitrogen; CRP, c-reactive protein; Hb, Hemoglobin.

Table S4 Multicollinearity analysis in the association analysis between cuWHH-WHR combined group and CMM

Variables	GVIF	Df	GVIF^(1/(2*Df))
cuWHH-WHR combined group	1.164	1	1.079

Age	1.574	1	1.254
Gender	2.272	1	1.507
Marital status	1.158	1	1.076
Education	1.148	1	1.071
Smoking	1.844	1	1.358
Drinking	1.295	1	1.138
SBP, mmHg	3.342	1	1.828
DBP, mmHg	2.614	1	1.617
UA, mg/dL	1.611	1	1.269
Scr, mg/dL	2.107	1	1.451
BUN, mg/dL	1.191	1	1.092
CRP, mg/dL	1.121	1	1.059
Cystatinc, mg/dL	1.388	1	1.178
Hb, g/dL	1.200	1	1.095
Hypertension	2.917	1	1.708
Chronic lung diseases	1.131	1	1.063
Liver disease	1.070	1	1.035
Kidney disease	1.141	1	1.068
Depression	1.000	1	1.000
Use of antihypertensive drugs	1.215	1	1.102
Use of hypoglycemic agents	1.215	1	1.102

GVIF, generalized variance inflation factor; Df, degrees of freedom; SBP, systolic blood pressure; DBP, diastolic blood pressure; UA, Serum uric acid; Scr, Serum Creatinine; BUN, Blood Urea Nitrogen; CRP, c-reactive protein; Hb, Hemoglobin.

Table S5 Schoenfeld residual test for the multivariate-adjusted Cox model in the original cohort

**Variable**	**chisq**	**df**	***P* value**
WHH-WHR	1.578	3	0.664
Combined group			
Age	2.589	1	0.108
Gender	0.124	1	0.724
Marital status	1.067	1	0.302
Education	1.651	1	0.199
Smoking	1.171	1	0.279
Drinking	0.212	1	0.645
SBP, mmHg	2.185	1	0.139
DBP, mmHg	0.023	1	0.880
UA, mg/dL	0.301	1	0.583
Scr, mg/dL	0.398	1	0.528
BUN, mg/dL	0.332	1	0.565

CRP, mg/dL	1.525	1	0.217
Cystatinc, mg/dL	3.139	1	0.076
Hb, g/dL	0.089	1	0.766
Hypertension	2.496	1	0.114
Chronic lung diseases	1.655	1	0.198
Liver disease	2.371	1	0.124
Kidney disease	1.778	1	0.182
Depression	0.706	1	0.401
Use of antihypertensive drugs	2.142	1	0.143
Use of hypoglycemic agents	1.395	1	0.238
GLOBAL	26.213	26	0.451

SBP, systolic blood pressure; DBP, diastolic blood pressure; UA, Serum uric acid; Scr, Serum Creatinine; BUN, Blood Urea Nitrogen; CRP, c-reactive protein; Hb, Hemoglobin.

Table S6 Schoenfeld residual test for the multivariate-adjusted Cox model in the cumulative exposure cohort

**Variable**	**chisq**	**df**	***P* value**
cuWHH-WHR Combined group	0.472	3	0.925
Age	0.010	1	0.920
Gender	0.228	1	0.633
Marital status	0.016	1	0.900
Education	0.246	1	0.620
Smoking	0.016	1	0.901
Drinking	0.888	1	0.346
SBP, mmHg	1.741	1	0.187
DBP, mmHg	0.111	1	0.739
UA, mg/dL	0.121	1	0.728
Scr, mg/dL	0.0820	1	0.775
BUN, mg/dL	0.0741	1	0.785
CRP, mg/dL	0.373	1	0.541
Cystatinc, mg/dL	0.423	1	0.516
Hb, g/dL	0.004	1	0.949
Hypertension	3.680	1	0.055
Chronic lung diseases	2.420	1	0.120
Liver disease	7.79 1	1	0.0053
Kidney disease	1.31 1	1	0.253
Depression	< 0.0001	1	1.000
Use of antihypertensive drugs	4.06 1	1	0.044
Use of hypoglycemic	2.28 1	1	0.131

agents			
GLOBAL	25.3	25	0.444

SBP, systolic blood pressure; DBP, diastolic blood pressure; UA, Serum uric acid; Scr, Serum Creatinine; BUN, Blood Urea Nitrogen; CRP, c-reactive protein; Hb, Hemoglobin.

Table S7 Baseline characteristics grouped according to the cuWHH-WHR combination

**Variables**	**Total**	**Group 1**	**Group 2**	**Group 3**	**Group 4**	***P* value**
n	4021	1182	829	829	1181	
CMM	181 (4.5)	19 (1.6)	50 (6)	16 (1.9)	96 (8.1)	＜ 0.001
Age, years	57.9 ± 8.4	57.9 ± 8.5	58.3 ± 8.3	57.2 ± 8.4	58.0 ± 8.4	0.037
Gender						＜ 0.001
Female	2207 (54.9)	583 (49.3)	643 (77.6)	264 (31.8)	717 (60.7)	
Male	1814 (45.1)	599 (50.7)	186 (22.4)	565 (68.2)	464 (39.3)	
Marital status						0.001
Married	3663 (91.1)	1086 (91.9)	727 (87.7)	756 (91.2)	1094 (92.6)	
Unmarried	358 (8.9)	96 (8.1)	102 (12.3)	73 (8.8)	87 (7.4)	
Education						< 0.001
Primary education	2827 (70.3)	841 (71.2)	622 (75)	537 (64.8)	827 (70)	
Secondary education	1110 (27.6)	316 (26.7)	191 (23)	280 (33.8)	323 (27.3)	
Higher education	84 (2.1)	25 (2.1)	16 (1.9)	12 (1.4)	31 (2.6)	
Smoking	13 (0.3)	2 (0.2)	5 (0.6)	3 (0.4)	3 (0.3)	< 0.001
Drinking	822 (20.4)	256 (21.7)	127 (15.3)	222 (26.8)	217 (18.4)	< 0.001
BMI, kg/m^2	23.7 ± 11.0	21.3 ± 2.8	25.2 ± 16.6	22.0 ± 3.1	26.3 ± 13.7	< 0.001
SBP, mmHg	128.0 ± 20.2	123.6 ± 18.8	129.6 ± 20.5	125.2 ± 18.8	133.3 ± 20.9	< 0.001
DBP, mmHg	74.9 ± 11.8	72.6 ± 11.4	75.4 ± 11.5	73.5 ± 11.5	77.8 ± 11.9	< 0.001
Waist circumference, cm	83.9 ± 12.1	76.5 ± 11.2	88.5 ± 7.8	78.4 ± 12.1	92.0 ± 8.7	< 0.001
Height, cm	157.8 ± 8.8	158.5 ± 8.0	154.6 ± 8.7	161.0 ± 8.2	157.2 ± 9.3	< 0.001
Weight, kg	58.7 ± 10.9	53.8 ± 9.2	59.3 ± 9.8	57.3 ± 10.2	64.3 ± 11.0	< 0.001

PLT, 10^9/L	212.0 ± 72.0	198.7 ± 64.7	205.2 ± 66.2	220.3 ± 78.3	224.2 ± 75.5	< 0.001
WBC, 10^9/L	6.2 ± 1.8	5.3 ± 1.3	5.2 ± 1.2	7.1 ± 1.9	7.1 ± 1.8	< 0.001
TG, mg/dL	103.5 (73.5, 152.2)	81.4 (62.2, 110.6)	98.2 (74.3, 135.4)	107.1 (74.3, 154.9)	144.3 (99.1, 212.4)	< 0.001
TC, mg/dL	193.4 ± 38.3	189.0 ± 34.3	202.2 ± 36.6	184.1 ± 37.6	198.1 ± 41.6	< 0.001
HDL-C, mg/dL	51.3 ± 15.2	61.3 ± 14.5	57.2 ± 14.0	45.0 ± 11.1	41.6 ± 10.4	< 0.001
LDL-C, mg/dL	115.9 ± 34.2	110.9 ± 29.6	125.0 ± 32.8	111.4 ± 34.9	117.5 ± 37.4	< 0.001
HbA1c, %	5.3 ± 0.8	5.0 ± 0.4	5.4 ± 0.9	5.0 ± 0.4	5.6 ± 1.1	< 0.001
FBG, mg/dL	109.5 ± 35.0	100.7 ± 16.5	112.3 ± 38.7	103.8 ± 24.9	120.3 ± 46.9	< 0.001
UA, mg/dL	4.3 ± 1.2	4.2 ± 1.1	4.1 ± 1.1	4.5 ± 1.3	4.6 ± 1.2	< 0.001
BUN, mg/dL	15.6 ± 4.3	15.9 ± 4.4	15.2 ± 4.2	15.7 ± 4.4	15.5 ± 4.1	0.002
Scr, mg/dL	0.8 ± 0.2	0.8 ± 0.2	0.7 ± 0.2	0.8 ± 0.2	0.8 ± 0.2	< 0.001
CRP, mg/L	0.9 (0.5, 1.9)	0.7 (0.4, 1.2)	0.9 (0.5, 1.7)	0.9 (0.5, 2.0)	1.4 (0.7, 2.7)	< 0.001
Hb, g/dL	14.4 ± 2.2	14.1 ± 2.2	14.1 ± 2.2	14.6 ± 2.2	14.6 ± 2.2	< 0.001
Cystatinc, mg/dL	1.0 ± 0.2	1.0 ± 0.2	1.0 ± 0.3	1.0 ± 0.2	0.9 ± 0.2	< 0.001
Hypertension	1392 (34.6)	293 (24.8)	319 (38.5)	217 (26.2)	563 (47.7)	< 0.001
Diabetes	626 (15.6)	91 (7.7)	145 (17.5)	78 (9.4)	312 (26.4)	< 0.001
Heart disease	72 (1.8)	17 (1.4)	19 (2.3)	19 (2.3)	17 (1.4)	0.258
Dyslipidemia	755 (18.8)	132 (11.2)	181 (21.8)	103 (12.4)	339 (28.7)	< 0.001
Chronic lung disease	327 (8.1)	100 (8.5)	59 (7.1)	81 (9.8)	87 (7.4)	0.156
Liver disease	134 (3.3)	46 (3.9)	32 (3.9)	21 (2.5)	35 (3)	0.261
Cancer	37 (0.9)	10 (0.8)	5 (0.6)	9 (1.1)	13 (1.1)	0.647
Kidney disease	230 (5.7)	79 (6.7)	40 (4.8)	51 (6.2)	60 (5.1)	0.217
Depression	40 (1.0)	10 (0.8)	13 (1.6)	9 (1.1)	8 (0.7)	0.23
Use of lipid-lowering drugs	772 (19.2)	141 (11.9)	186 (22.4)	103 (12.4)	342 (29)	< 0.001
Use of	555 (13.8)	85 (7.2)	128 (15.4)	75 (9)	267 (22.6)	<

antihypertensive drugs						0.001
Use of hypoglycemic agents	115 (2.9)	6 (0.5)	28 (3.4)	4 (0.5)	77 (6.5)	< 0.001

Normally distributed data are presented as mean ± SD, non-normally distributed data are presented as medians (interquartile range). Categorical variables are presented as frequency (%). CMM, Cardiometabolic multimorbidity; BMI, body mass index; SBP, systolic blood pressure; DBP, diastolic blood pressure; PLT, Platelets; WBC, white blood cell; TG, triglycerides; HDL-C, High Density Lipoprotein-Cholesterol; TC, total cholesterol; LDL-C, low-density lipoprotein cholesterol; HbA1c, glycated hemoglobin; FPG, fasting plasma glucose; UA, Serum uric acid; Scr, Serum Creatinine; BUN, Blood Urea Nitrogen; CRP, c-reactive protein; Hb, Hemoglobin

Table S8 Association between quartiles of WHH, WHR, cuWHH, and cuWHR with CMM

**Characteristic**	**N (Incidence rate)**	**Model1**		**Model2**		**Model3**	
		**HR (95% CI)**	***P* value**	**HR (95% CI)**	***P* value**	**HR (95% CI)**	***P* value**
**WHH**							
**Q1**	50(3.4)	Ref	-	Ref	-	Ref	-
**Q2**	79(5.3)	1.37 (0.87-2.15)	0.17	1.31 (0.83-2.05)	0.246	1.34 (0.85-2.1)	0.209
**Q3**	95(6.4)	1.99 (1.31-3.03)	0.001	1.76 (1.15-2.69)	0.009	1.73 (1.13-2.65)	0.012
**Q4**	131(8.9)	6.88 (4.77-9.93)	<0.001	5.67 (3.88-8.3)	<0.001	4.5 (3.04-6.67)	<0.001
***P* for trend**			<0.001		<0.001		<0.001
**WHR**							
**Q1**	33(2.2)	Ref	-	Ref	-	Ref	-
**Q2**	45(3)	1.66(1.12-2.28)	0.01	1.62 (1.14-2.31)	0.008	1.58 (1.11-2.26)	0.012
**Q3**	65(4.4)	1.93 (1.37-2.71)	<0.001	1.88 (1.33-2.65)	<0.001	1.63 (1.15-2.3)	0.007
**Q4**	212(14.3)	2.71 (1.95-3.75)	<0.001	2.57 (1.85-3.58)	<0.001	1.99 (1.41-2.8)	<0.001

***P* for trend**			<0.001		<0.001		<0.001
**cuWHH**							
**Q1**	16 (1.6)	Ref	-	Ref	-	Ref	-
**Q2**	19 (1.9)	1.19 (0.61-2.32)	0.606	1.13 (0.58-2.2)	0.719	1.2 (0.61-2.34)	0.601
**Q3**	37 (3.7)	2.34 (1.3-4.2)	0.005	2.02 (1.11-3.67)	0.022	2.04 (1.12-3.72)	0.021
**Q4**	109 (10.8)	7.18 (4.25-12.13)	<0.001	5.76 (3.34-9.96)	<0.001	4.63 (2.63-8.13)	<0.001
***P* for trend**			<0.001		<0.001		<0.001
**cuWHR**							
**Q1**	24 (2.4)	Ref	-	Ref	-	Ref	-
**Q2**	45 (4.5)	1.9 (1.16-3.12)	0.012	1.89 (1.15-3.11)	0.013	1.84 (1.12-3.03)	0.017
**Q3**	45 (4.5)	1.9 (1.16-3.11)	<0.001	1.92 (1.17-3.15)	0.011	1.72 (1.04-2.84)	0.036
**Q4**	67 (6.7)	2.86 (1.79-4.56)	<0.001	2.98 (1.86-4.78)	<0.001	2.35 (1.45-3.82)	0.001
***P* for trend**			<0.001		<0.001		0.002

HR, Hazard ratio; CI, Confidence interval; WHH, Waist-to-Height-Hemoglobin A1c; WHR, White blood cell-to-high-density lipoprotein cholesterol ratio.

Model I: unadjusted

Model II: Adjusted age, gender, marital status, education

Model III: Adjusted age, gender, marital status, education, smoking, drinking, SBP, DBP, UA, Scr, BUN, CRP, Cystatinc, Hb, hypertension, chronic lung diseases, liver disease, kidney disease, depression, use of antihypertensive drugs, use of hypoglycemic agents.

Table S9 Threshold effect analysis of WHR for CMM risk with optimal cut-off value

**WHR**	**Adjusted HR (95% CI)**	***P* value**
Linear	1.061 (1.041-1.082)	< 0.001
Cox proportional hazards model with segmentation		

Cutpoint	5.418	
WHR< 5.418	1.193 (1.063-1.34)	0.0028
WHR≥ 5.418	1.052 (1.026-1.078)	< 0.001
Concordance	0.739 (se = 0.013)	
P for log-likelihood ratio	Chisq=4.2186	0.03998

WHR, White blood cell-to-high-density lipoprotein cholesterol ratio; HR, Hazard ratio; CI, Confidence interval.

Adjusted age, gender, marital status, education, smoking, drinking, SBP, DBP, UA, Scr, BUN, CRP, Cystatinc, Hb, hypertension, chronic lung diseases, liver disease, kidney disease, depression, use of antihypertensive drugs, use of hypoglycemic agents.

Table S10 DeLong test of the AUC of the cumulative incidence rate at 9 years

**Model Comparison**	**AUC**	**New AUC**	***P* value**
Base model vs. Base+WHH model	0.728	0.751	＜0.0001
Base model vs. Base+WHR model	0.728	0.738	0.0037
Base model vs. Base+WHH +WHR model	0.728	0.758	＜0.0001
Base+WHH model vs. Base+WHH+WHR model	0.751	0.758	0.0148
Base+WHR model vs. Base+WHH+WHR model	0.738	0.758	＜0.0001

WHH, Waist-to-Height-Hemoglobin A1c; WHR, White blood cell-to-high-density lipoprotein cholesterol ratio; AUC, Area Under the Curve.

Table S11 Predictive performance of models for CMM risk at 4, 7, and 9 years of follow-up in the original cohort

**Follow up**	**Comparison**	**Model1 AUC**	**Model2 AUC**	**Improvement**	**NRI (95%CI)**	**IDI (95%CI)**
4-year follow up	Base vs Base+WHH	0.742	0.779	0.037	0.3438(0.0545,0.6572)	0.006(-0.005,0.026)
	Base vs Base+WHH+WHR	0.742	0.798	0.056	0.4403(0.1727,0.7417)	0.004(-0.010,0.021)
	Base vs Base+WHR	0.742	0.765	0.023	0.1390(-0.0628,0.5471)	-0.001(-0.006,0.005)
	Base+WHH vs Base+WHH+WHR	0.779	0.798	0.019	0.1243(-0.1536,0.4924)	-0.002(-0.008,0.004)
	Base+WHR vs Base+WHH+WHR	0.765	0.798	0.033	0.3089(-0.0086,0.6246)	0.005(-0.006,0.022)
7-year follow up	Base vs Base+WHH	0.748	0.773	0.025	0.3583(0.1267,0.5728)	0.011(0.000,0.026)
	Base vs Base+WHH+WHR	0.748	0.781	0.033	0.3416(0.1401,0.5405)	0.020(0.004,0.042)
	Base vs Base+WHR	0.748	0.759	0.011	0.0440(-0.1040,0.2945)	0.010(0.000,0.022)
	Base+WHH vs	0.773	0.781	0.008	-0.0140(-0.14	0.009(0.000

	Base+WHH+WHR				73,0.2523)	,0.027)
	Base+WHR vs Base+WHH+WHR	0.759	0.781	0.022	0.2855(0.0985,0.5346)	0.010(0.001,0.029)
9-year follow up	Base vs Base+WHH	0.728	0.751	0.023	0.3316(0.0979,0.5171)	0.017(0.008,0.029)
	Base vs Base+WHH+WHR	0.728	0.758	0.03	0.2792(0.1047,0.4757)	0.021(0.011,0.033)
	Base vs Base+WHR	0.728	0.738	0.01	0.0416(-0.1239,0.2490)	0.005(0.001,0.01)
	Base+WHH vs Base+WHH+WHR	0.751	0.758	0.007	-0.0485(-0.1475,0.2077)	0.004(0.000,0.011)
	Base+WHR vs Base+WHH+WHR	0.738	0.758	0.02	0.2559(0.0796,0.4477)	0.016(0.008,0.029)

WHH, Waist-to-Height-Hemoglobin A1c; WHR, White blood cell-to-high-density lipoprotein cholesterol ratio; AUC, Area Under the Curve; NRI, Net Reclassification Index; IDI, Integrated Discrimination Improvement.

Table S12 Predictive performance of models for CMM risk at 5-year-follow-up in in the cumulative exposure cohort

Follow up	Comparison	Model 1AUC	Model 2AUC	Improvement	NRI (95%CI)	IDI (95%CI)
5-year follow up	Base vs Base+cuWHH	0.7209	0.7616	0.0407	0.022(0.006,0.041)	0.184(0.093,0.277)
	Base vs Base+cuWHH+cuWHR	0.7209	0.7625	0.0416	0.093(-0.004,0.17)	0.014(0.005,0.032)
	Base vs Base+cuWHR	0.7209	0.7336	0.0127	0.219(0.118,0.298)	0.028(0.011,0.055)
	Base+cuWHH vs Base+cuWHH+cuWHR	0.7616	0.7625	0.0009	0.025(-0.063,0.135)	0.006(0,0.019)
	Base+cuWHR vs Base+cuWHH+cuWHR	0.7336	0.7625	0.0289	0.133(0.051,0.228)	0.014(0.002,0.031)

WHH, Waist-to-Height-Hemoglobin A1c; WHR, White blood cell-to-high-density lipoprotein cholesterol ratio; AUC, Area Under the Curve; NRI, Net Reclassification Index; IDI, Integrated Discrimination Improvement.

Table S13 Interaction of WHH and WHR on CMM risk

Interactive indices	Interactive effects (95% CI)		
	Model I	Model II	Model III
Additive effect			
RERI	0.79 (-0.77, 2.18)	0.8 (-1.37, 2.69)	-0.36 (-2.61, 0.89)
AP	0.15 (-0.11, 0.34)	0.14 (-0.16, 0.36)	-0.12 (-0.69, 0.27)
SI	1.22 (0.87, 1.69)	1.2 (0.83, 1.74)	0.84 (0.47, 1.51)

Multiplicative effect	0.8 (0.48, 1.33)	0.73 (0.39, 1.35)	0.67 (0.32, 1.38)

Model I: unadjusted

Model II: Adjusted age, gender, marital status, education

Model III: Adjusted age, gender, marital status, education, smoking, drinking, SBP, DBP, UA, Scr, BUN, CRP, Cystatinc, Hb, hypertension, chronic lung diseases, liver disease, kidney disease, depression, use of antihypertensive drugs, use of hypoglycemic agents.

RERI, Relative Excess Risk due to Interaction(RERI); AP, Attributable Proportion due to Interaction; SI, Synergy Index.

Table S14 Interaction of cuWHH and cuWHR on CMM risk

Interactive indices	Interactive effects (95% CI)		
	**Model I**	**Model II**	**Model III**
Additive effect			
RERI	1.18 (-1, 3.27)	1.22 (-0.98, 3.31)	0.46 (-2.63, 2.7)
AP	0.23 (-0.13, 0.47)	0.28 (-0.14, 0.56)	0.14 (-0.56, 0.57)
SI	1.39 (0.84, 2.3)	1.56 (0.79, 3.1)	1.25 (0.5, 3.14)
Multiplicative effect	1.13 (0.54, 2.39)	1.48 (0.6, 3.66)	1.17 (0.4, 3.47)

Model I: unadjusted

Model II: Adjusted age, gender, marital status, education

Model III: Adjusted age, gender, marital status, education, smoking, drinking, SBP, DBP, UA, Scr, BUN, CRP, Cystatinc, Hb, hypertension, chronic lung diseases, liver disease, kidney disease, depression, use of antihypertensive drugs, use of hypoglycemic agents.

RERI, Relative Excess Risk due to Interaction(RERI); AP, Attributable Proportion due to Interaction; SI, Synergy Index.

Table S15 Sensitivity analysis in the original cohort using complete case analysis (without multiple imputation)

**Characteristic**	**N (Incidence rate)**	**Model1**		**Model2**		**Model3**	
		**HR (95% CI)**	***P* value**	**HR (95% CI)**	***P* value**	**HR (95% CI)**	***P* value**
**WHH**	-	1.0044 (1.0039-1.0048)	<0.001	1.004 (1.004-1.005)	<0.001	1.0037 (1.003-1.004)	<0.001
**WHR**	-	1.08 (1.06-1.09)	<0.001	1.09 (1.07-1.11)	<0.001	1.06 (1.04-1.08)	<0.001
**WHH-WHR combined groups**							
**Group1**	40(2.2)	Ref	-	Ref	-	Ref	-
**Group2**	43(3.5）	1.66 (1.08-2.55)	0.022	1.69 (1.1-2.61)	0.017	1.62 (1.05-2.5)	0.029

**Group3**	97(7.9)	3.74 (2.59-5.41)	<0.001	3.21 (2.21-4.67)	<0.001	2.94 (2.01-4.28)	<0.001
**Group4**	175(10.8)	5.25 (3.73-7.41)	<0.001	4.34 (3.06-6.15)	<0.001	3.29 (2.28-4.74)	<0.001
***P* for trend**			<0.001		<0.001		<0.001

HR, Hazard ratio; CI, Confidence interval; WHH, Waist-to-Height-Hemoglobin A1c; WHR, White blood cell-to-high-density lipoprotein cholesterol ratio.

Group 1: low WHH + low WHR, Group 2: low WHH + high WHR, Group 3: high WHH + low WHR, Group 4: high WHH + high WHR.

Model I: unadjusted

Model II: Adjusted age, gender, marital status, education

Model III: Adjusted age, gender, marital status, education, smoking, drinking, SBP, DBP, UA, Scr, BUN, CRP, Cystatinc, Hb, hypertension, chronic lung diseases, liver disease, kidney disease, depression, use of antihypertensive drugs, use of hypoglycemic agents.

Table S16 Sensitivity analysis in the original cohort by excluding participants who developed CMM in 2013

**Characteristic**	**N (Incidence rate)**	**Model1**		**Model2**		**Model3**	
		**HR (95% CI)**	***P* value**	**HR (95% CI)**	***P* value**	**HR (95% CI)**	***P* value**
**WHH**	-	1.0043 (1.0038-1.0048)	<0.001	1.0043 (1.0038-1.0048)	<0.001	1.004 (1.0034-1.0046)	<0.001
**WHR**	-	1.09 (1.07-1.1)	<0.001	1.07 (1.05-1.09)	<0.001	1.07 (1.05-1.1)	<0.001
**WHH-WHR combined groups**							
**Group1**	39 (2.1)	Ref	-	Ref	-	Ref	-
**Group2**	35 (2.9)	1.38 (0.88-2.18)	0.165	1.41 (0.89-2.23)	0.142	1.35 (0.85-2.14)	0.199
**Group3**	84 (6.9)	3.34 (2.28-4.88)	<0.001	2.86 (1.94-4.21)	<0.001	2.63 (1.79-3.88)	<0.001
**Group4**	155 (9.7)	4.8 (3.38-6.82)	<0.001	3.94 (2.76-5.64)	<0.001	3 (2.06-4.36)	<0.001

***P* for trend**			<0.001		<0.001		<0.001

HR, Hazard ratio; CI, Confidence interval; WHH, Waist-to-Height-Hemoglobin A1c; WHR, White blood cell-to-high-density lipoprotein cholesterol ratio.

Group 1: low WHH + low WHR, Group 2: low WHH + high WHR, Group 3: high WHH + low WHR, Group 4: high WHH + high WHR.

Model I: unadjusted

Model II: Adjusted age, gender, marital status, education

Model III: Adjusted age, gender, marital status, education, smoking, drinking, SBP, DBP, UA, Scr, BUN, CRP, Cystatinc, Hb, hypertension, chronic lung diseases, liver disease, kidney disease, depression, use of antihypertensive drugs, use of hypoglycemic agents.

Table S17 Sensitivity analysis in the original cohort excluding participants with baseline diabetes, CVD, or stroke

**Characteristic**	**N (Incidence rate)**	**Model1**		**Model2**		**Model3**	
		**HR (95% CI)**	***P* value**	**HR (95% CI)**	***P* value**	**HR (95% CI)**	***P* value**
**WHH**	-	1.004 (1.0029-1.005)	<0.001	1.0037 (1.0024-1.0049)	<0.01	1.0036 (1.0023-1.0049)	<0.001
**WHR**	-	1.11 (1.06-1.17)	<0.001	1.11 (1.05-1.17)	<0.001	1.11 (1.05-1.17)	<0.001
**WHH-WHR combined groups**							
**Group1**	31 (1.9)	Ref	-	Ref	-	Ref	-
**Group2**	29 (2.7)	1.47 (0.89-2.44)	0.136	1.48 (0.89-2.47)	0.133	1.43 (0.86-2.38)	0.173
**Group3**	51 (5.2)	2.8 (1.79-4.38)	<0.001	2.55 (1.61-4.02)	<0.001	2.5 (1.58-3.96)	<0.001
**Group4**	69 (6.2)	3.4 (2.22-5.19)	<0.001	2.84 (1.84-4.4)	<0.001	2.65 (1.7-4.13)	<0.001
***P* for trend**			<0.001		<0.001		<0.001

HR, Hazard ratio; CI, Confidence interval; WHH, Waist-to-Height-Hemoglobin A1c; WHR, White blood cell-to-high-density lipoprotein cholesterol ratio.

Group 1: low WHH + low WHR, Group 2: low WHH + high WHR, Group 3: high WHH + low

WHR, Group 4: high WHH + high WHR.

Model I: unadjusted

Model II: Adjusted age, gender, marital status, education

Model III: Adjusted age, gender, marital status, education, smoking, drinking, SBP, DBP, UA, Scr, BUN, CRP, Cystatinc, Hb, hypertension, chronic lung diseases, liver disease, kidney disease, depression, use of antihypertensive drugs, use of hypoglycemic agents.

Table S18 Sensitivity analysis in the original cohort excluding participants with baseline dyslipidemia

**Characteristic**	**N (Incidence rate)**	**Model1**		**Model2**		**Model3**	
		**HR (95% CI)**	***P* value**	**HR (95% CI)**	***P* value**	**HR (95% CI)**	***P* value**
**WHH**	-	1.0041 (1.0028-1.0053)	<0.001	1.0038 (1.0024-1.0052)	<0.001	1.0035 (1.0018-1.0052)	<0.001
**WHR**	-	1.12 (0.99-1.28)	0.077	1.13 (1-1.28)	0.045	1.1 (0.96-1.25)	0.181
**WHH-WHR combined groups**							
**Group1**	20 (1.5)	Ref	-	Ref	-	Ref	-
**Group2**	9 (1.9)	1.27 (0.58-2.8)	0.55	1.39 (0.63-3.07)	0.419	1.31 (0.59-2.9)	0.508
**Group3**	35 (5.3)	3.59 (2.07-6.23)	<0.001	3.3 (1.87-5.8)	<0.001	2.86 (1.6-5.12)	0.001
**Group4**	18 (4.8)	3.29 (1.74-6.22)	<0.001	3.28 (1.71-6.29)	0.001	2.65 (1.34-5.23)	0.007
***P* for trend**			<0.001		<0.001		<0.001

HR, Hazard ratio; CI, Confidence interval; WHH, Waist-to-Height-Hemoglobin A1c; WHR, White blood cell-to-high-density lipoprotein cholesterol ratio.

Group 1: low WHH + low WHR, Group 2: low WHH + high WHR, Group 3: high WHH + low WHR, Group 4: high WHH + high WHR.

Model I: unadjusted

Model II: Adjusted age, gender, marital status, education

Model III: Adjusted age, gender, marital status, education, smoking, drinking, SBP, DBP, UA, Scr, BUN, CRP, Cystatinc, Hb, hypertension, chronic lung diseases, liver disease, kidney disease, depression, use of antihypertensive drugs, use of hypoglycemic agents.

Table S19 Sensitivity analysis in the original cohort excluding participants with baseline hypertension

Characteristic	N (Incidence rate)	Model1		Model2		Model3	
		HR (95% CI)	*P* value	HR (95% CI)	*P* value	HR (95% CI)	*P* value
**WHH**	-	1.006 (1.005-1.007)	<0.001	1.0062 (1.005-1.0074)	<0.001	1.0057 (1.0043-1.0071)	<0.001
**WHR**	-	1.08 (1.06-1.1)	<0.001	1.05 (1.03-1.07)	<0.001	1.05 (1.03-1.08)	<0.001
**WHH-WHR combined groups**							
**Group1**	21 (1.5)	Ref	-	Ref	-	Ref	-
**Group2**	25 (2.8)	1.86 (1.04-3.33)	0.038	1.94 (1.08-3.48)	0.027	1.83 (1.02-3.3)	0.045
**Group3**	41 (5.5)	3.63 (2.14-6.14)	<0.001	3.46 (2.03-5.9)	<0.001	3.08 (1.8-5.28)	<0.001
**Group4**	61 (7.4)	5.01 (3.05-8.22)	<0.001	5.03 (3.05-8.28)	<0.001	4.17 (2.48-7.01)	<0.001
***P* for trend**			<0.001		<0.001		<0.001

HR, Hazard ratio; CI, Confidence interval; WHH, Waist-to-Height-Hemoglobin A1c; WHR, White blood cell-to-high-density lipoprotein cholesterol ratio.

Group 1: low WHH + low WHR, Group 2: low WHH + high WHR, Group 3: high WHH + low WHR, Group 4: high WHH + high WHR.

Model I: unadjusted

Model II: Adjusted age, gender, marital status, education

Model III: Adjusted age, gender, marital status, education, smoking, drinking, SBP, DBP, UA, Scr, BUN, CRP, Cystatinc, Hb, hypertension, chronic lung diseases, liver disease, kidney disease, depression, use of antihypertensive drugs, use of hypoglycemic agents.

Table S20 Sensitivity analysis in the original cohort excluding participants using hypoglycemic agents, antihypertensive, or lipid-lowering medications at baseline

Characteristic	N (Incidence rate)	Model1		Model2		Model3	
		HR (95% CI)	*P* value	HR (95% CI)	*P* value	HR (95% CI)	*P* value
**WHH**	-	1.0041 (1.0031-1.00	<0.001	1.004 (1.003-1.00	<0.001	1.0039 (1.0028-1.00	<0.001

		5)		5)		5)	
**WHR**	-	1.12 (1.08-1.17)	<0.001	1.13 (1.08-1.17)	<0.001	1.11 (1.06-1.16)	<0.001
**WHH-WHR combined groups**							
**Group1**	28 (1.7)	Ref	-	Ref	-	Ref	-
**Group2**	34 (3.3)	1.9 (1.15-3.14)	0.013	1.99 (1.2-3.29)	0.008	1.93 (1.16-3.2)	0.012
**Group3**	49 (5.3)	3.07 (1.93-4.88)	<0.001	2.95 (1.84-4.72)	<0.001	2.8 (1.74-4.51)	<0.001
**Group4**	72 (6.9)	4.09 (2.64-6.33)	<0.001	4.03 (2.59-6.26)	<0.001	3.64 (2.32-5.72)	<0.001
***P* for trend**			<0.001		<0.001		0.001

HR, Hazard ratio; CI, Confidence interval; WHH, Waist-to-Height-Hemoglobin A1c; WHR, White blood cell-to-high-density lipoprotein cholesterol ratio.

Group 1: low WHH + low WHR, Group 2: low WHH + high WHR, Group 3: high WHH + low WHR, Group 4: high WHH + high WHR.

Model I: unadjusted

Model II: Adjusted age, gender, marital status, education

Model III: Adjusted age, gender, marital status, education, smoking, drinking, SBP, DBP, UA, Scr, BUN, CRP, Cystatinc, Hb, hypertension, chronic lung diseases, liver disease, kidney disease, depression, use of antihypertensive drugs, use of hypoglycemic agents.

Table S21 Sensitivity analysis in the original cohort excluding participants with extreme values

**Characteristic**	**N (Incidence rate)**	**Model1**		**Model2**		**Model3**	
		**HR (95% CI)**	***P* value**	**HR (95% CI)**	***P* value**	**HR (95% CI)**	***P* value**
**WHH**	-	1.0114 (1.0099-1.0129)	<0.001	1.0102 (1.0086-1.0118)	<0.001	1.0083 (1.0064-1.0102)	<0.001
**WHR**	-	1.08 (1.06-1.1)	<0.001	1.08 (1.06-1.1)	<0.001	1.06 (1.04-1.09)	<0.001

**WHH-WHR combined groups**							
**Group1**	39 (2.2)	Ref	-	Ref	-	Ref	-
**Group2**	39 (3.3)	1.53 (0.98-2.38)	0.062	1.59 (1.02-2.48)	0.043	1.53 (0.98-2.39)	0.062
**Group3**	88 (7.3)	3.41 (2.34-4.97)	<0.001	2.92 (1.99-4.28)	<0.001	2.72 (1.85-4.01)	<0.001
**Group4**	151(9.8)	4.67 (3.28-6.63)	<0.001	3.85 (2.69-5.52)	<0.001	3.1 (2.13-4.5)	<0.001
***P* for trend**			<0.001		<0.001		0.001

HR, Hazard ratio; CI, Confidence interval; WHH, Waist-to-Height-Hemoglobin A1c; WHR, White blood cell-to-high-density lipoprotein cholesterol ratio.

Group 1: low WHH + low WHR, Group 2: low WHH + high WHR, Group 3: high WHH + low WHR, Group 4: high WHH + high WHR.

Model I: unadjusted

Model II: Adjusted age, gender, marital status, education

Model III: Adjusted age, gender, marital status, education, smoking, drinking, SBP, DBP, UA, Scr, BUN, CRP, Cystatinc, Hb, hypertension, chronic lung diseases, liver disease, kidney disease, depression, use of antihypertensive drugs, use of hypoglycemic agents.

Table S22 Sensitivity analysis in the cumulative exposure cohort using complete case analysis (without multiple imputation)

Characteristic	N (Incidence rate)	Model1		Model2		Model3	
		HR (95% CI)	*P* value	HR (95% CI)	*P* value	HR (95% CI)	*P* value
**Cu-WHH**	-	1.0026 (1.0023-1.003)	<0.001	1.06 (1.04-1.09)	<0.001	1.0019 (1.0013-1.0026)	<0.001
**Cu-WHR**	-	1.06 (1.04-1.08)	<0.001	1.0025 (1.002-1.0029)	<0.001	1.05 (1.02-1.08)	0.001
**Cu-WHH-WHR combined groups**							
**Group1**	39 (2.1)	Ref	-	Ref	-	Ref	-
**Group2**	35 (2.9)	3.84	<0.0	3.08	<0.0	2.92	0.00

		(2.26-6.51)	01	(1.69-5.6)	01	(1.39-6.15)	5
**Group3**	84 (6.9)	1.2 (0.62-2.33)	0.591	0.88 (0.38-2.03)	0.772	0.96 (0.36-2.56)	0.932
**Group4**	155 (9.7)	5.23 (3.2-8.56)	<0.001	3.86 (2.2-6.76)	<0.001	3.3 (1.62-6.7)	0.001
***P* for trend**			<0.001		<0.001		<0.001

HR, Hazard ratio; CI, Confidence interval; WHH, Waist-to-Height-Hemoglobin A1c; WHR, White blood cell-to-high-density lipoprotein cholesterol ratio.

Group 1: low WHH + low WHR, Group 2: low WHH + high WHR, Group 3: high WHH + low WHR, Group 4: high WHH + high WHR.

Model I: unadjusted

Model II: Adjusted age, gender, marital status, education

Model III: Adjusted age, gender, marital status, education, smoking, drinking, SBP, DBP, UA, Scr, BUN, CRP, Cystatinc, Hb, hypertension, chronic lung diseases, liver disease, kidney disease, depression, use of antihypertensive drugs, use of hypoglycemic agents.

Table S23 Sensitivity analysis in the cumulative exposure cohort by excluding participants who developed CMM in 2018

**Characteristic**	**N (Incidence rate)**	**Model1**		**Model2**		**Model3**	
		**HR (95% CI)**	***P* value**	**HR (95% CI)**	***P* value**	**HR (95% CI)**	***P* value**
**Cu-WHH**		1.0023 (1.0014-1.0032)	<0.001	1.0021 (1.0011-1.003)	<0.001	1.0022 (1.001-1.0033)	0.002
**Cu-WHR**		1.05 (1.01-1.1)	0.035	1.07 (1.02-1.11)	0.009	1.06 (1.01-1.11)	0.04
**Cu-WHH-WHR combined groups**							
**Group1**	3 (0.3)	Ref	-	Ref	-	Ref	-
**Group2**	11 (1.4)	5.44 (1.52-19.51)	0.014	4.4 (1.2-16.04)	0.035	4.19 (1.14-15.41)	0.05
**Group3**	6 (0.7)	2.85 (0.71-11.41)	0.147	3.51 (0.87-14.12)	0.091	3.32 (0.82-13.47)	0.117
**Group4**	20 (1.8)	7.09 (2.11-23.85)	0.003	6.55 (1.92-22.3)	0.006	5.66 (1.62-19.7)	0.017
***P* for trend**			0.002		0.002		0.007

HR, Hazard ratio; CI, Confidence interval; WHH, Waist-to-Height-Hemoglobin A1c; WHR,

White blood cell-to-high-density lipoprotein cholesterol ratio.

Group 1: low WHH + low WHR, Group 2: low WHH + high WHR, Group 3: high WHH + low WHR, Group 4: high WHH + high WHR.

Model I: unadjusted

Model II: Adjusted age, gender, marital status, education

Model III: Adjusted age, gender, marital status, education, smoking, drinking, SBP, DBP, UA, Scr, BUN, CRP, Cystatinc, Hb, hypertension, chronic lung diseases, liver disease, kidney disease, depression, use of antihypertensive drugs, use of hypoglycemic agents.

Table S24 Sensitivity analysis in the cumulative exposure cohort excluding participants with baseline diabetes, CVD, or stroke

**Characteristic**	**N (Incidence rate)**	**Model1**		**Model2**		**Model3**	
		**HR (95% CI)**	***P* value**	**HR (95% CI)**	***P* value**	**HR (95% CI)**	***P* value**
**Cu-WHH**		1.0027 (1.0019-1.0034)	<0.001	1.0025 (1.0017-1.0033)	<0.001	1.0026 (1.0017-1.0034)	<0.001
**Cu-WHR**		1.06 (1.03-1.09)	<0.001	1.06 (1.03-1.09)	<0.001	1.06 (1.03-1.1)	<0.001
**Cu-WHH-WHR combined groups**							
**Group1**	16 (1.5)	Ref	-	Ref	-	Ref	-
**Group2**	23 (3.5)	2.36 (1.25-4.46)	0.01	2.21 (1.15-4.25)	0.02	2.11 (1.09-4.1)	0.03
**Group3**	10 (1.4)	0.92 (0.42-2.02)	0.828	0.95 (0.43-2.1)	0.892	0.93 (0.42-2.06)	0.853
**Group4**	47 (5.5)	3.78 (2.14-6.66)	<0.001	3.52 (1.97-6.29)	<0.001	3.4 (1.88-6.15)	<0.001
***P* for trend**			<0.001		<0.001		<0.001

HR, Hazard ratio; CI, Confidence interval; WHH, Waist-to-Height-Hemoglobin A1c; WHR, White blood cell-to-high-density lipoprotein cholesterol ratio.

Group 1: low WHH + low WHR, Group 2: low WHH + high WHR, Group 3: high WHH + low WHR, Group 4: high WHH + high WHR.

Model I: unadjusted

Model II: Adjusted age, gender, marital status, education

Model III: Adjusted age, gender, marital status, education, smoking, drinking, SBP, DBP, UA, Scr, BUN, CRP, Cystatinc, Hb, hypertension, chronic lung diseases, liver disease, kidney disease, depression, use of antihypertensive drugs, use of hypoglycemic agents.

Table S25 Sensitivity analysis in the cumulative exposure cohort excluding participants with baseline dyslipidemia

Characteristic	N (Incidence rate)	Model1		Model2		Model3	
		HR (95% CI)	*P* value	HR (95% CI)	*P* value	HR (95% CI)	*P* value
**Cu-WHH**		1.0026 (1.0021-1.0032)	<0.001	1.0026 (1.002-1.0031)	<0.001	1.0023 (1.0016-1.003)	<0.001
**Cu-WHR**		1.05 (1.02-1.07)	0.002	1.05 (1.02-1.08)	<0.001	1.04 (1.01-1.07)	0.006
**Cu-WHH-WHR combined groups**							
**Group1**	16 (1.5)	Ref	-	Ref	-	Ref	-
**Group2**	35 (5.4)	3.61 (2-6.52)	<0.001	3.45 (1.88-6.32)	<0.001	3.06 (1.66-5.66)	0.001
**Group3**	13 (1.8)	1.17 (0.56-2.44)	0.668	1.24 (0.59-2.59)	0.57	1.17 (0.56-2.44)	0.686
**Group4**	46 (5.5)	3.65 (2.07-6.45)	<0.001	3.58 (2.01-6.36)	<0.001	2.94 (1.62-5.34)	0.001
***P* for trend**			0.001		0.001		0.015

HR, Hazard ratio; CI, Confidence interval; WHH, Waist-to-Height-Hemoglobin A1c; WHR, White blood cell-to-high-density lipoprotein cholesterol ratio.

Group 1: low WHH + low WHR, Group 2: low WHH + high WHR, Group 3: high WHH + low WHR, Group 4: high WHH + high WHR.

Model I: unadjusted

Model II: Adjusted age, gender, marital status, education

Model III: Adjusted age, gender, marital status, education, smoking, drinking, SBP, DBP, UA, Scr, BUN, CRP, Cystatinc, Hb, hypertension, chronic lung diseases, liver disease, kidney disease, depression, use of antihypertensive drugs, use of hypoglycemic agents.

Table S26 Sensitivity analysis in the cumulative exposure cohort excluding participants with baseline hypertension

Characteristic	N (Incidence rate)	Model1		Model2		Model3	
		HR (95% CI)	*P* value	HR (95% CI)	*P* value	HR (95% CI)	*P* value
**Cu-WHH**		1.0027 (1.0021-1.0033)	<0.001	1.0027 (1.002-1.0033)	<0.001	1.0026 (1.0018-1.0034)	<0.001

**Cu-WHR**		1.04 (1-1.07)	0.033	1.05 (1.01-1.08)	0.008	1.04 (1-1.08)	0.035
**Cu-WHH-WHR combined groups**							
**Group1**	13 (1.5)	Ref	-	Ref	-	Ref	-
**Group2**	28 (5.5)	3.82 (1.98-7.38)	<0.001	3.68 (1.87-7.22)	<0.001	3.32 (1.68-6.56)	0.001
**Group3**	7 (1.1)	0.78 (0.31-1.96)	0.598	0.79 (0.31-1.99)	0.617	0.76 (0.3-1.92)	0.563
**Group4**	34 (5.5)	3.84 (2.03-7.27)	<0.001	3.93 (2.06-7.47)	<0.001	3.4 (1.75-6.6)	0.001
***P* for trend**			0.003		0.003		0.004

HR, Hazard ratio; CI, Confidence interval; WHH, Waist-to-Height-Hemoglobin A1c; WHR, White blood cell-to-high-density lipoprotein cholesterol ratio.

Group 1: low WHH + low WHR, Group 2: low WHH + high WHR, Group 3: high WHH + low WHR, Group 4: high WHH + high WHR.

Model I: unadjusted

Model II: Adjusted age, gender, marital status, education

Model III: Adjusted age, gender, marital status, education, smoking, drinking, SBP, DBP, UA, Scr, BUN, CRP, Cystatinc, Hb, hypertension, chronic lung diseases, liver disease, kidney disease, depression, use of antihypertensive drugs, use of hypoglycemic agents.

Table S27 Sensitivity analysis in the cumulative exposure cohort excluding participants using hypoglycemic agents, antihypertensive, or lipid-lowering medications at baseline

**Characteristic**	**N (Incidence rate)**	**Model1**		**Model2**		**Model3**	
		**HR (95% CI)**	***P* value**	**HR (95% CI)**	***P* value**	**HR (95% CI)**	***P* value**
**Cu-WHH**		1.0027 (1.002-1.0033)	<0.001	1.0026 (1.0019-1.0033)	<0.001	1.0026 (1.0018-1.0033)	<0.001
**Cu-WHR**		1.04 (1.01-1.07)	0.004	1.05 (1.02-1.08)	0.001	1.04 (1.01-1.08)	0.007
**Cu-WHH-WHR combined groups**							
**Group1**	15 (1.5)	Ref	-	Ref	-	Ref	-
**Group2**	31 (5)	3.49 (1.88-6.46)	<0.001	3.39 (1.8-6.38)	<0.001	3.07 (1.61-5.83)	0.001

**Group3**	13 (1.8)	1.25 (0.6-2.63)	0.553	1.32 (0.62-2.78)	0.474	1.25 (0.59-2.64)	0.565
**Group4**	40 (5)	3.53 (1.95-6.39)	<0.001	3.56 (1.96-6.46)	<0.001	3.17 (1.71-5.86)	<0.001
***P* for trend**			0.001		0.001		0.005

HR, Hazard ratio; CI, Confidence interval; WHH, Waist-to-Height-Hemoglobin A1c; WHR, White blood cell-to-high-density lipoprotein cholesterol ratio.

Group 1: low WHH + low WHR, Group 2: low WHH + high WHR, Group 3: high WHH + low WHR, Group 4: high WHH + high WHR.

Model I: unadjusted

Model II: Adjusted age, gender, marital status, education

Model III: Adjusted age, gender, marital status, education, smoking, drinking, SBP, DBP, UA, Scr, BUN, CRP, Cystatinc, Hb, hypertension, chronic lung diseases, liver disease, kidney disease, depression, use of antihypertensive drugs, use of hypoglycemic agents.

Table S28 Sensitivity analysis in the cumulative exposure cohort excluding participants with extreme values

**Characteristic**	**N (Incidence rate)**	**Model1**		**Model2**		**Model3**	
		**HR (95% CI)**	***P* value**	**HR (95% CI)**	***P* value**	**HR (95% CI)**	***P* value**
**Cu-WHH**		1.0033 (1.0027-1.0039)	<0.001	1.003 (1.0023-1.0036)	<0.001	1.0024 (1.0016-1.0031)	<0.001
**Cu-WHR**		1.05 (1.03-1.08)	<0.001	1.06 (1.03-1.09)	<0.001	1.05 (1.02-1.08)	0.002
**Cu-WHH-WHR combined groups**							
**Group1**	19 (1.7)	Ref	-	Ref	-	Ref	-
**Group2**	45 (5.6)	3.34 (1.95-5.71)	<0.001	2.88 (1.67-4.98)	<0.001	2.56 (1.48-4.44)	0.001
**Group3**	15 (1.9)	1.09 (0.55-2.15)	0.8	1.2 (0.61-2.37)	0.604	1.14 (0.57-2.25)	0.716
**Group4**	82 (7.3)	4.4 (2.67-7.25)	<0.001	3.84 (2.31-6.37)	<0.001	3.08 (1.83-5.19)	<0.001
***P* for trend**			<0.001		<0.001		<0.001

HR, Hazard ratio; CI, Confidence interval; WHH, Waist-to-Height-Hemoglobin A1c; WHR, White blood cell-to-high-density lipoprotein cholesterol ratio.

Group 1: low WHH + low WHR, Group 2: low WHH + high WHR, Group 3: high WHH + low

WHR, Group 4: high WHH + high WHR.

Model I: unadjusted

Model II: Adjusted age, gender, marital status, education

Model III: Adjusted age, gender, marital status, education, smoking, drinking, SBP, DBP, UA, Scr, BUN, CRP, Cystatinc, Hb, hypertension, chronic lung diseases, liver disease, kidney disease, depression, use of antihypertensive drugs, use of hypoglycemic agents.

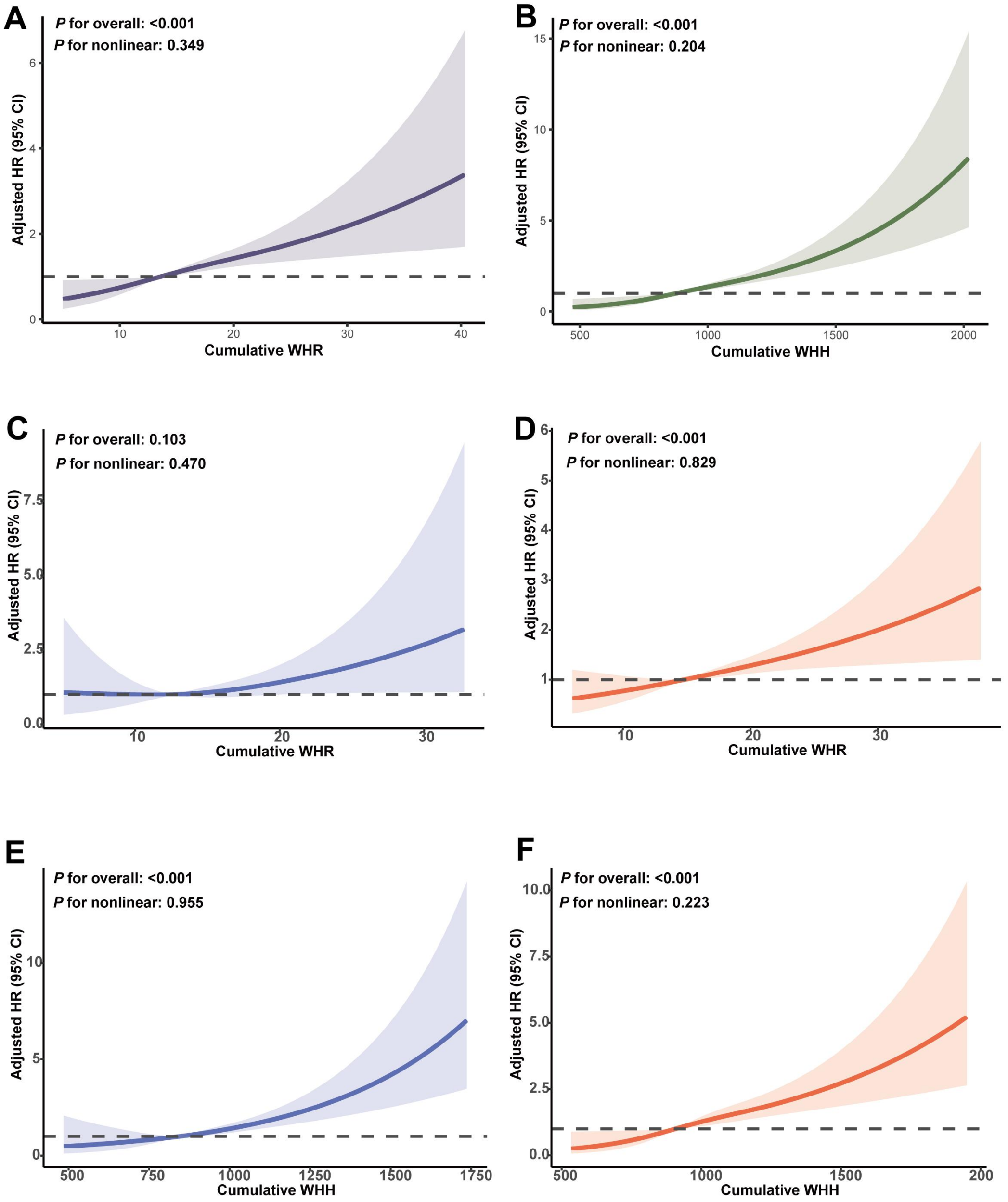

Fig. S1. Restricted cubic spline analysis of cuWHH and cuWHR with CMM risk. (A) cuWHR; (B) cuWHH; (C) WHR RCS stratified by low cuWHH; (D) WHR RCS stratified by high cuWHH; (E) WHH RCS stratified by low cuWHR; (F) WHH RCS stratified by high cuWHR.

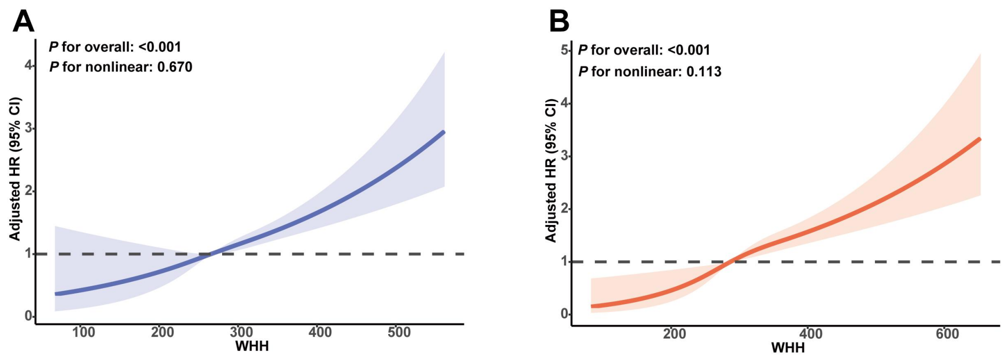

Fig. S2. Restricted cubic spline analysis of WHR with CMM risk stratified by the optimal cut-off value of 5.418.

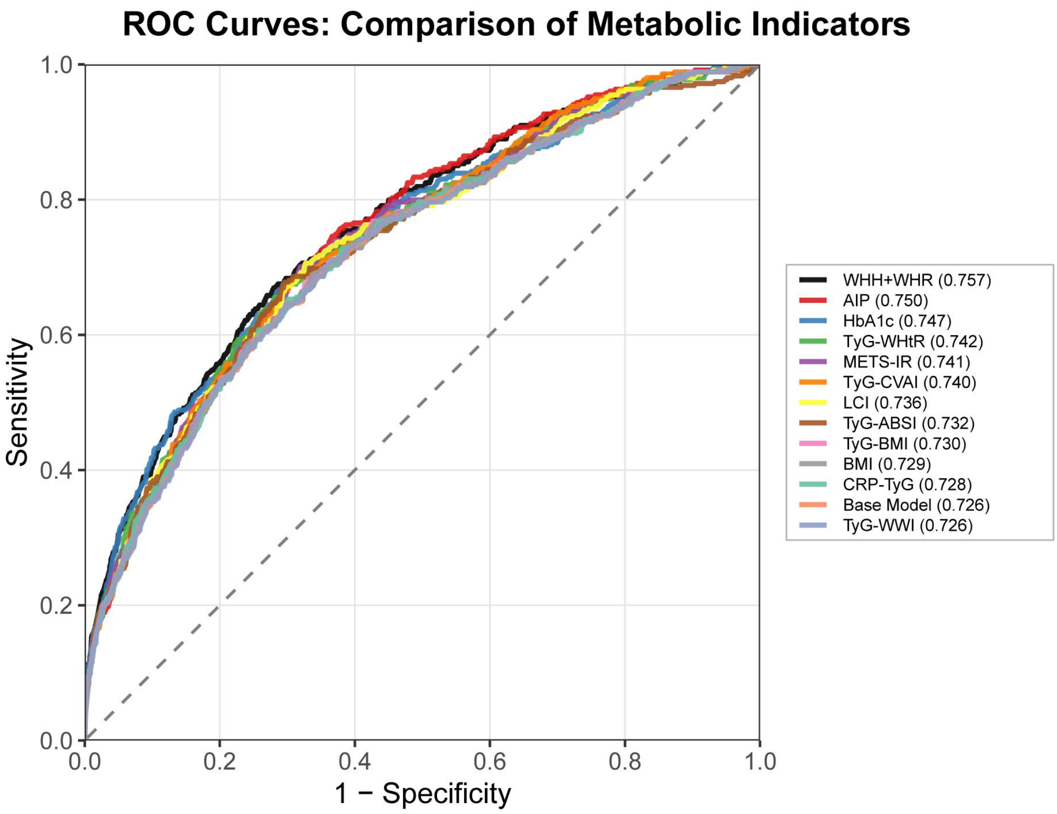

Fig. S3. Comparison of ROC curves for CMM risk prediction between WHH-WHR combined model and other metabolic indices.

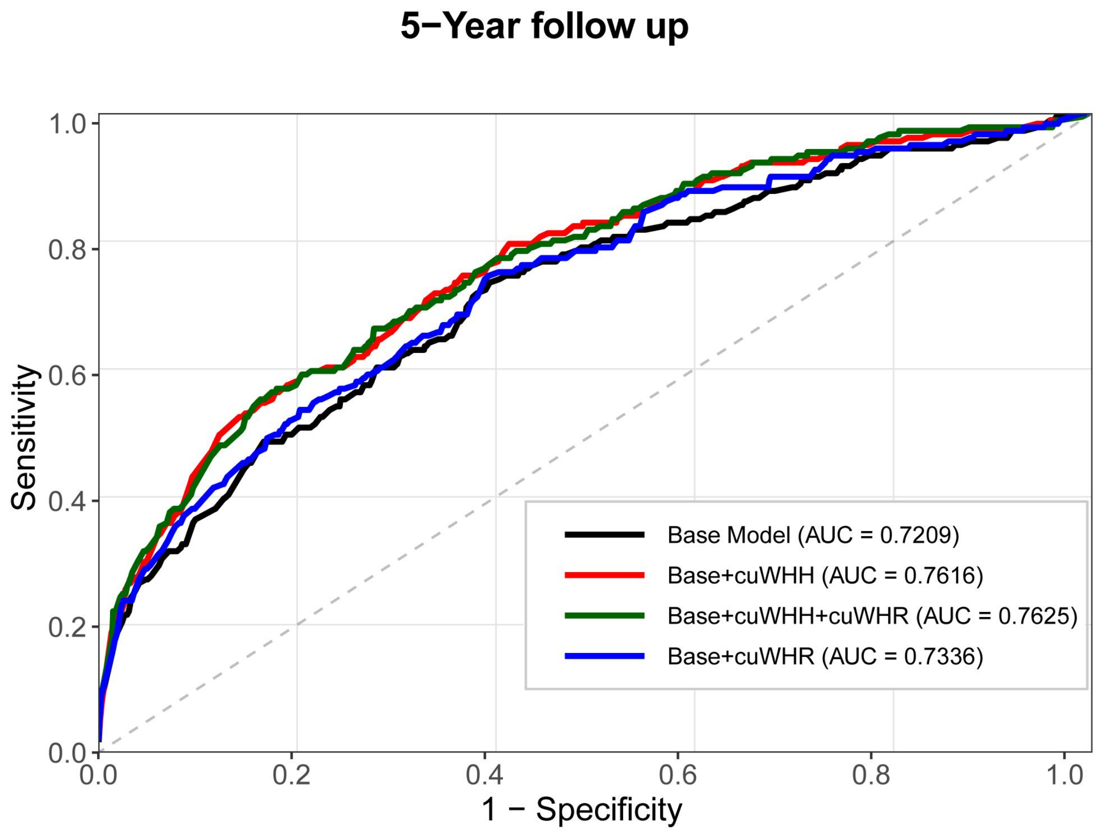

Fig. S4. Predictive performance of cumulative exposure models for CMM risk.

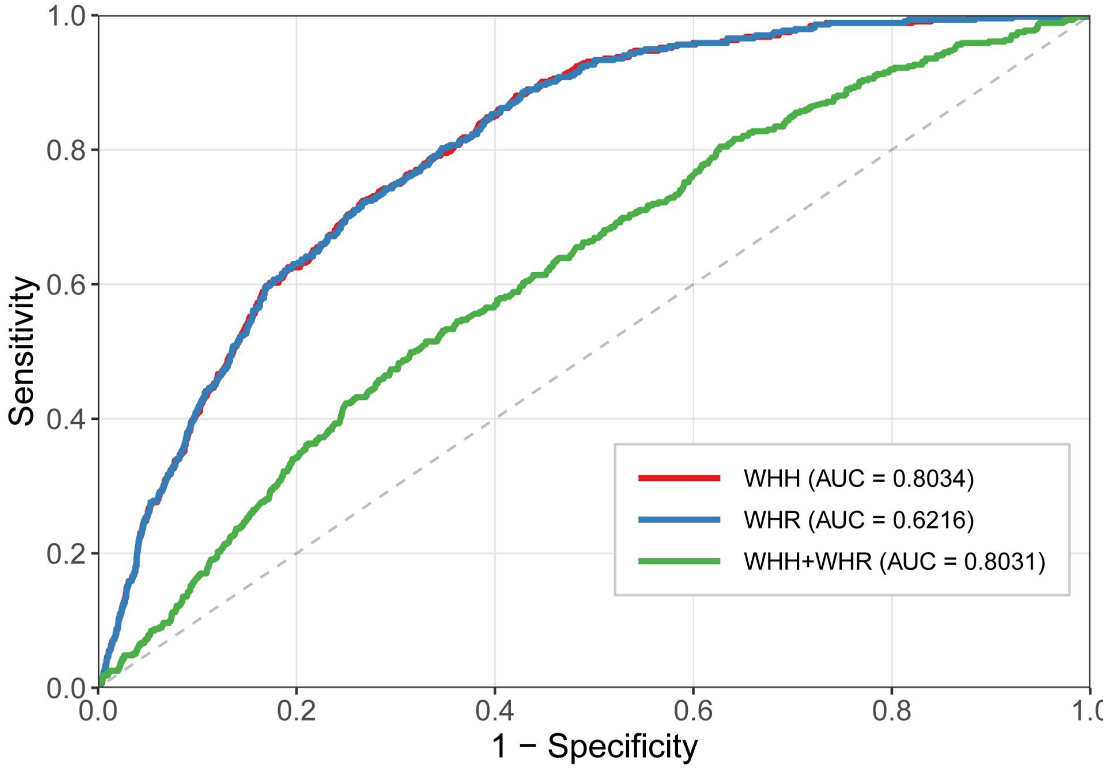

Fig. S5. ROC Curves for WHH and WHR in Predicting CMM (NHANES).

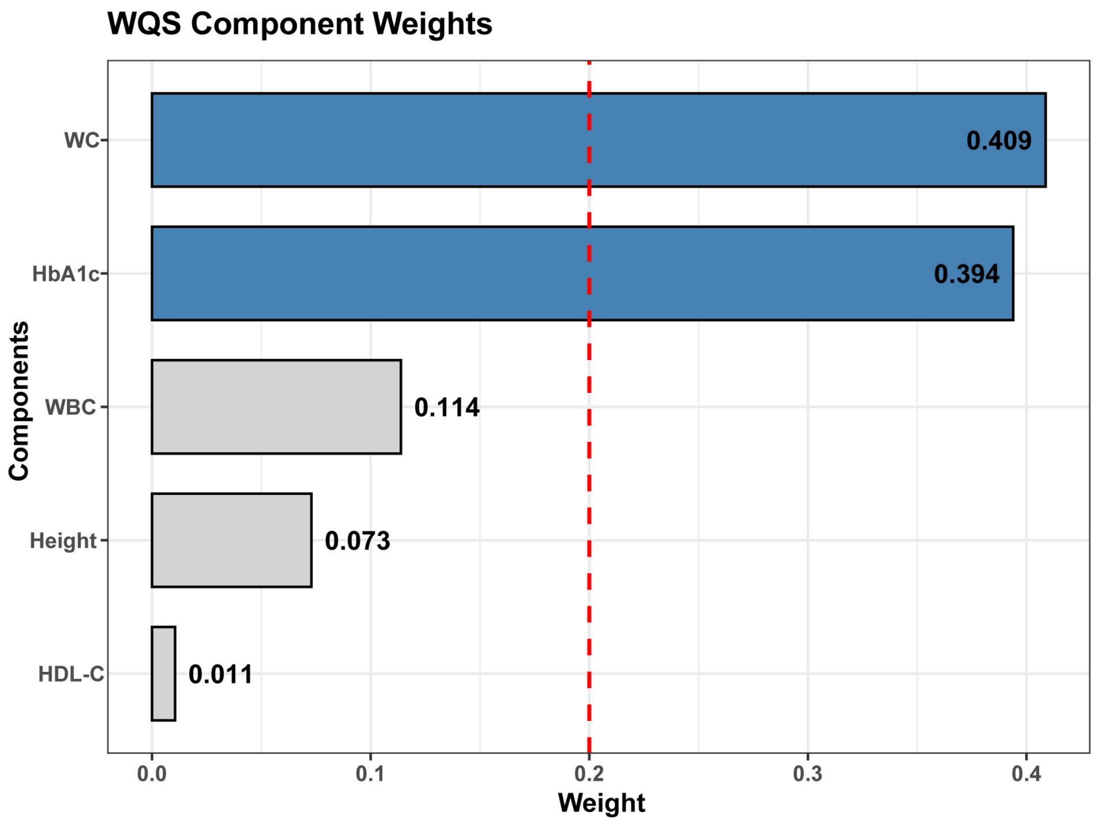

Fig. S6. Weighted quantile sum (WQS) regression analysis of WHH and WHR components. Bar plot showing contribution weights of each component (waist circumference, HbA1c, WBC, height, HDL-C).

Subgroups	Event/Total(%)	HR (95%CI)	*P* value	*P* for Interaction
**Age**				**0.841**
<60	340/48 (6.9)	2.48 (1.15~5.34)	0.02	
≥60	841/48 (10)	2.25 (1~5.06)	0.049	
**Gender**				**0.254**
Female	717/70 (9.8)	2.93 (1.37~6.25)	0.005	
Male	464/26 (5.6)	1.79 (0.75~4.26)	0.19	
**Drink**				**0.634**
Yes	235/14 (6)	1.36 (0.38~4.91)	0.64	
No	946/82 (8.7)	2.61 (1.4~4.84)	0.002	
**Smoke**				**0.509**
Yes	393/26 (6.6)	2.15 (0.84~5.5)	0.111	
No	788/70 (8.9)	2.63 (1.3~5.3)	0.007	
**BMI**				**0.113**
<24	340/24 (7.1)	3.41 (1.69~6.88)	0.001	
≥24	841/72 (8.6)	1.45 (0.57~3.66)	0.434	
**Dyslipidemia**				**0.388**
Yse	340/50 (14.7)	3.78 (1.08~13.26)	0.038	
No	841/46 (5.5)	2.07 (1.08~3.94)	0.027	
**Diabetes**				**0.53**
Yse	312/48 (15.4)	1.07 (0.85~1.35)	0.577	
No	869/48 (5.5)	2.7 (1.44~5.05)	0.002	
**Hypertension**				**0.62**
Yse	563/62 (11)	2.71 (1.08~6.78)	0.033	
No	618/34 (5.5)	2.34 (1.13~4.83)	0.022	
**Heart disease**				**0.405**
Yse	17/1 (5.9)	1 (0.06~15.99)	0.999	
No	1164/95 (8.2)	2.46 (1.39~4.34)	0.002	
**Kidney disease**				**0.235**
Yse	60/5 (8.3)	0.99 (0.16~6.11)	0.994	
No	1121/91 (8.1)	2.59 (1.43~4.69)	0.002	
**Liver disease**				**0.485**
Yse	35/7 (20)	13.11 (0.95~181.69)	0.055	
No	1146/89 (7.8)	2.31 (1.3~4.08)	0.004	
**Chronic lung diseases**				**0.205**
Yse	87/11 (12.6)	1.18 (0.31~4.51)	0.805	
No	1094/85 (7.8)	2.86 (1.53~5.35)	0.001	
**Use of lipid−lowering drugs**				**0.277**
Yes	342/51 (14.9)	4.05 (1.15~14.28)	0.03	
No	839/45 (5.4)	2.04 (1.07~3.89)	0.031	
**Use of antihypertensive drugs**				**0.197**
Yes	276/44 (16.5)	10.1 (1.29~79.26)	0.028	
No	914/52 (5.7)	1.84 (1~3.38)	0.049	
**Use of hypoglycemic agents**				**0.52**
Yes	77/23 (29.9)	2.12 (0.23~19.52)	0.507	
No	1104/73 (6.6)	2.35 (1.32~4.2)	0.004	

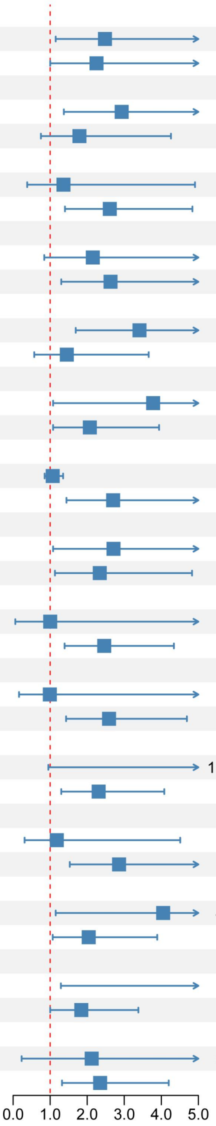

Fig. S7. Subgroup analysis for the association between high cuWHH+high cuWHR group and CMM risk.
